# Tumor‐Infiltrating Immune Cells Are More Abundant in Lung Metastases From Colorectal Cancer Than in Paired Primary Tumors and Their Prognostic Value Depends on Adjuvant Chemotherapy

**DOI:** 10.1002/cam4.71739

**Published:** 2026-03-20

**Authors:** Danyil Kuznyecov, Christina Siesing, Halla Vidarsdottir, Jakob Eberhard, Hans Brunnström, Karin Leandersson, Karin Jirström

**Affiliations:** ^1^ Department of Clinical Sciences Lund, Division of Oncology and Therapeutic Pathology Lund University Lund Sweden; ^2^ Department of Clinical Genetics, Pathology and Molecular Diagnostics Skåne University Hospital Lund Sweden; ^3^ Department of Haematology, Oncology and Radiation Physics Skåne University Hospital Lund Sweden; ^4^ Department of Surgery Landspitali University Hospital Reykjavik Iceland; ^5^ Department of Clinical Sciences Lund, Division of Pathology Lund University Lund Sweden; ^6^ Department of Translational Medicine, Cancer Immunology Lund University Malmö Sweden

**Keywords:** adjuvant treatment, B cells, colorectal cancer, lung metastases, PD‐L1, prognosis, T cells, tertiary lymphoid‐like structures

## Abstract

**Introduction:**

The immune microenvironment plays a crucial role in the progression of colorectal cancer (CRC), but its variation across primary tumors and metastatic sites remains incompletely understood. In this study, we compared the composition and prognostic significance of the immune microenvironment in lung metastases, paired primary tumors, and liver metastases from a retrospective cohort of 216 patients with metastatic CRC who underwent pulmonary metastasectomy in a single Swedish institution.

**Methods:**

Immunohistochemistry was applied on tissue microarrays to evaluate CD3^+^, CD8^+^, CD20^+^, FoxP3^+^ immune cells, PD‐L1^+^ immune cells and tumor cells, and tertiary lymphoid‐like structures (TLLS).

**Results:**

Lung metastases exhibited significantly higher infiltration of all immune cell subsets compared to both primary tumors and liver metastases. FoxP3^+^ cell density was lower in liver metastases than in primary tumors. Immune cell infiltration in lung metastases was largely unaffected by neoadjuvant chemotherapy, except for lower FoxP3^+^ cell infiltration in treated cases. None of the immune markers bore prognostic significance in the overall analysis. However, high infiltration of FoxP3^+^, CD20^+^, and PD‐L1^+^ immune cells, and presence of TLLSs in lung metastases were associated with improved overall survival in patients receiving adjuvant chemotherapy, with a significant prognostic treatment interaction. None of the immune markers were prognostic in primary tumors or liver metastases.

**Conclusion:**

These findings underscore a distinct, more immunologically active immune microenvironment in CRC lung metastases compared to primary tumors and liver metastases, that suggestedly also predicts response to adjuvant chemotherapy. Immune profiling of lung metastases may thus pave the way for improved personalized treatment of these patients.

## 1 Introduction

CRC ranks as the third most common cancer globally and the second leading cause of cancer‐related mortality [1]. It contributes to roughly 10% of all annual cancer diagnoses and deaths [1]. At diagnosis, 20% of patients present with metastatic disease, and an additional 25% of those initially diagnosed with localized disease will subsequently develop metastases [2, 3]. Metastases predominantly affect the liver (20%–70%) and lungs (10%–20%) [4, 5], and CRC is the most frequent indication for both hepatic and pulmonary metastasectomy (PM) [6]. PM is considered feasible and safe, with low perioperative mortality [7]. However, it is performed with curative intent in approximately 4% of patients with synchronous and 14% of patients with metachronous lung metastases [8].

During the onset of CRC, the immune response identifies and targets tumor‐specific and tumor‐associated antigens, thereby inhibiting the progression of CRC [4]. Nevertheless, CRC can alter the tumor microenvironment (TME), facilitating cancer proliferation and progression by modulating angiogenesis, invasion, and immune infiltration, thus creating an immunosuppressive and inflammatory setting [4].

The liver and lung microenvironments are distinct metastatic niches, with demonstrated differences in the immune landscape between the primary tumor and metastatic sites in CRC [9]. Understanding the composition of the immune microenvironment in both primary and metastatic CRC (mCRC) is crucial for predicting patient outcomes and guiding treatment strategies [10]. Numerous studies have characterized the immune landscape in primary CRC, and the prognostic or predictive significance of various immune cell subsets [11, 12]. The immune landscape in mCRC has also been delineated [13], but few studies have made comparisons between the immune landscape in paired primary and metastatic sites in individual patients [14]. Therefore, this study aimed to evaluate the composition and prognostic significance of the immune microenvironment in lung metastases, paired primary tumors, and liver metastases in a retrospective cohort of 216 mCRC patients treated with curative intent.

## 2 Materials and Methods

### 2.1 Study Cohort

This retrospective, single‐institution study included all patients who underwent PM for mCRC with curative intent at Skåne University Hospital, Lund, between January 1, 2000, and December 31, 2014 [15]. From the pathology records, 224 patients were identified, excluding eight where surgery was performed without curative intent. A total of 216 patients underwent 288 surgical procedures, 159 underwent a single PM, while 42 and 15 patients, respectively, were surgically treated two or three times due to recurrent lung metastases [15]. Clinical data, including demographics, tumor staging, diagnostics, imaging, disease‐free interval (DFI), surgical details, metastasis characteristics, and oncologic treatment, were collected from hospital records, with specific blood/serum analyses within 2 months before PM. Elevated CEA was determined as > 5 μg/L and elevated CRP was > 10 mg/L. A total of 29 patients (14.5%) received neoadjuvant chemotherapy before primary tumor surgery, with 25 undergoing chemoradiotherapy for rectal cancer, and 77 patients (40%) received adjuvant therapy, including 15 who had both neoadjuvant and adjuvant chemotherapy. Prior to PM, 23 patients (11%) had neoadjuvant treatment, while 99 (50%) received adjuvant therapy, 9 of whom had both. Data on oncologic treatment and recurrence were unavailable for 13 patients.

The diagnosis of lung metastases was based on morphology, comparison with the primary tumor, and, in some cases, immunohistochemistry (IHC) [15]. Patients were typically investigated with computed tomography scans and discussed at a multidisciplinary tumor board. Synchronous metastasis was defined as diagnosed within 6 months of the primary tumor. DFI was defined as the time from curative resection of the primary tumor to surgery for the first lung metastasis, or, in case of prior liver resection, the time from liver surgery to first PM. Time to recurrence was from PM to first documented recurrence. Follow‐up time was measured from the first PM, with all patients followed until death or for at least 5 years.

### 2.2 Tissue Microarray Construction and Immunohistochemistry

Tissue microarray (TMA) blocks were constructed from lung metastases, primary tumors, and liver metastases using a semiautomated arraying device (TMArrayer, Pathology Devices, Westminster, MD, USA) [15]. Duplicate 1 mm cores were extracted from representative, well‐fixed, non‐necrotic regions. Cores were obtained from lung metastases in all 216 cases, from primary tumors in 174 cases (81%), and from liver metastases in 50 cases (23%). In cases with multiple resected lung or liver metastases, cores were taken from each metastasis.

All IHC stainings were performed on 4 μm thick TMA sections. For analysis of immune cells, the sections were automatically pretreated using the PT‐link system and stained on an Autostainer Plus (Agilent Technologies, Santa Clara, CA, USA). The following antibodies were used: anti‐CD3 (polyclonal, rabbit, ready‐to‐use, product code IR503, Dako, Glostrup, Denmark), anti‐PD‐L1 (clone E1L3N, rabbit, 1:100, Cell Signaling Technology Inc., Danvers, MA, USA), anti‐CD20 (HPA14341, rabbit, 1:200, Atlas Antibodies AB, Stockholm, Sweden), anti‐FoxP3 (clone 236A/E7, mouse, 1:200, Abcam, Cambridge, UK), and anti‐CD8 (C8/144B, mouse, 1:50, product M7103, Dako, Glostrup, Denmark).

Mismatch repair (MMR) protein analysis was performed using the Ventana Benchmark Ultra system with the following ready‐to‐use clones: MLH1 (M1), MSH2 (G219‐1129), MSH6 (SP93), and PMS2 (A16‐4), all supplied by Roche. Detection was done using the Optiview Detection Kit (Ventana Medical Systems Inc., Tucson, AZ, USA), with amplification for MLH1. Pretreatment times for these antibodies were 56, 32, 32, and 64 min at 100°C, respectively, all at pH 8.5.

IHC analyses were carried out by a board‐certified pathologist (DK) in a blinded manner, and scoring was performed using a conventional light microscope. Staining intensity was not considered. Double stainings were performed for CD3/CD8 and CD3/CD20, while the remaining IHC markers were assessed using single staining. FoxP3/CD3 double staining (FoxP3^+^CD3^+^) was also evaluated separately using double IHC to confirm FoxP3 expression in T‐cells (Figure S1A). Slides were also scanned at 40 × magnification using a Hamamatsu NanoZoomer S360 digital slide scanner (Hamamatsu Photonics, Hamamatsu City, Japan).

Cores were excluded from analysis if technical artifacts rendered them unreadable or if cancer cells were absent. Among the 216 patients, CD3/CD20 double staining could be evaluated in lung metastases from 208 cases, in both lung metastases and paired primary tumors from 161 cases, and in lung metastases, primary tumors, and liver metastases from 44 cases. For CD3/CD8 double staining, the corresponding numbers were 207, 160, and 43 cases; for FoxP3: 206, 159, and 43 cases; and for PD‐L1: 206, 158, and 43 cases, respectively. The fraction of CD3^+^ immune cells and the number of TLLSs were assessed in the CD3/CD20 double staining.

The fractions of CD3^+^ and CD8^+^ lymphocytes were evaluated separately, using a semiquantitative method and classified as 0 (< 1%), 1 (1%–10%), 2 (11%–25%), or 3 (≥ 26%). PD‐L1 expression in tumor cells (PD‐L1^TC^) was assessed by calculating the percentage of tumor cells with partial or complete membranous staining of any intensity. In immune cells (PD‐L1^IC^), both membranous and cytoplasmic staining were considered. The percentage of stained cells in both compartments was assessed semiquantitatively with the following values: 1 (0%–1%), 2 (2%–9%), 3 (10%–49%), and 4 (50%–100%). Expression of FoxP3 and CD20 was also evaluated semiquantitatively, based on the total number of stained cells, with scores assigned as 0 (0 cells), 1 (1–5 cells), 2 (6–10 cells), 3 (11–20 cells), 4 (21–50 cells), and 5 (≥ 51 cells). If discrepancies were observed between the values of the duplicate cores, the highest value was selected.

There is currently no consensus on the definition of tertiary lymphoid structures (TLS)/TLLS [16, 17]. We have chosen to use a more unrestricted definition where TLLSs were defined as lymphoid infiltrates, based on CD3 and CD20 staining, comprising 20 to more than 60 lymphoid cells, organized in a diffuse or nodular pattern with varying degrees of structural organization. In cases of discrepancy between duplicate cores, the core exhibiting the highest TLLS count was selected as the definitive value.

Deficient MMR status was defined as the loss of staining in cancer cells for any of the four MMR proteins (MSH2, MSH6, MLH1, and PMS2) across all evaluated cores. Proficient MMR status was defined as positive staining in any of the two cores. MMR deficiency was seen in 6 out of 200 lung metastases (3.0%) and 6 out of 146 primary tumors (3.8%). A total of five cases exhibited discrepant MMR status between the first lung metastasis and the primary tumor, including two synchronous and three metachronous metastases.

### 2.3 Statistical Analyses

All statistical analyses were performed using IBM SPSS Statistics version 29.0 (IBM, Armonk, NY, USA). Statistical tests were two‐sided, with *p*‐values < 0.05 considered significant. Graphs were generated using SPSS or GraphPad Prism version 10 (GraphPad Software, La Jolla, CA, USA). Wilcoxon signed‐rank test was used to assess the difference in immune cell density between primary tumors and lung or liver metastases. Mann–Whitney *U* test was used to compare lung metastases subgroups stratified by neoadjuvant treatment status. Crosstabulation and the χ^2^ test were applied to evaluate associations between immune cell expression within the TME in primary tumors and lung metastases, and clinicopathological characteristics. Survival was calculated from the time of the first lung metastasis to death or last follow‐up. The Kaplan–Meier method was employed to estimate overall survival (OS), with differences assessed by the log‐rank test. Univariable and multivariable Cox proportional hazards models were used to estimate hazard ratios (HRs) and evaluate the impact of the presence of stained cells on survival.

## 3 Results

### 3.1 Distribution of Immune Markers in Lung Metastases, Primary Tumors and Liver Metastases

**FIGURE 1:**
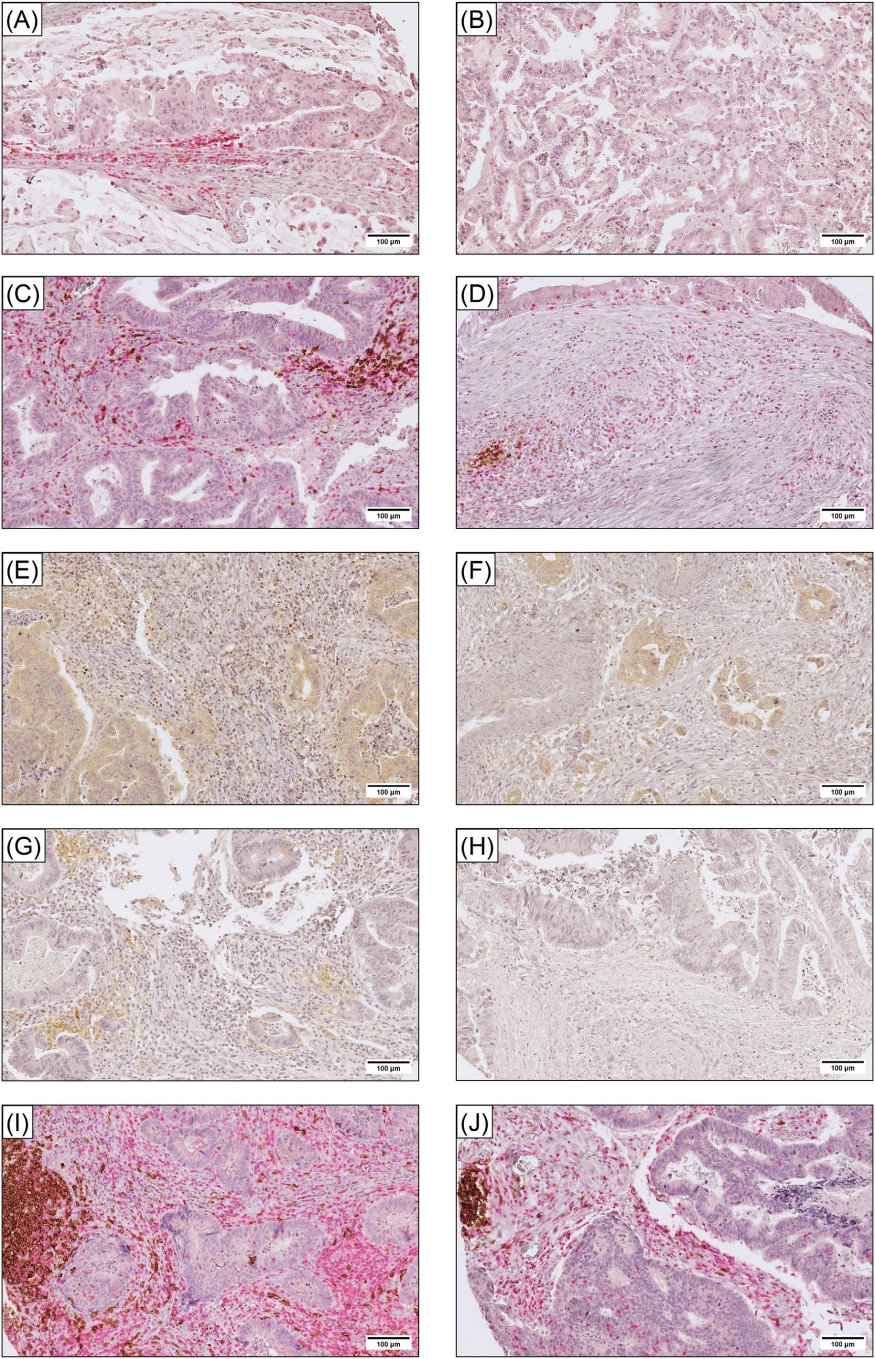
Representative IHC images of lung metastases and paired primary tumors. Double staining for CD3/CD8: (A) the lung metastasis showed a score of 3 for CD3 and 2 for CD20, and (B) the primary tumor scored 0 for both markers. Double staining for CD3/CD20: (C) the lung metastasis scored 3 for CD3 and 5 for CD20; and (D) the primary tumor scored 2 for CD3 and 3 for CD20. Single staining for FoxP3, where scores were 5 and 2 in the lung metastasis and primary tumor, respectively (E, F). Single staining for PD‐L1, where the lung metastasis scored 1 in tumor cells and 3 in immune cells, while the primary tumor scored 1 in both compartments (G, H); CD3/CD20 double staining highlighting TLLS, where 1 TLLS was observed in both the lung metastasis and the primary tumor (I, J). All images were captured at 20 × magnification. Scale bar: 100 μm.

**FIGURE 2:**
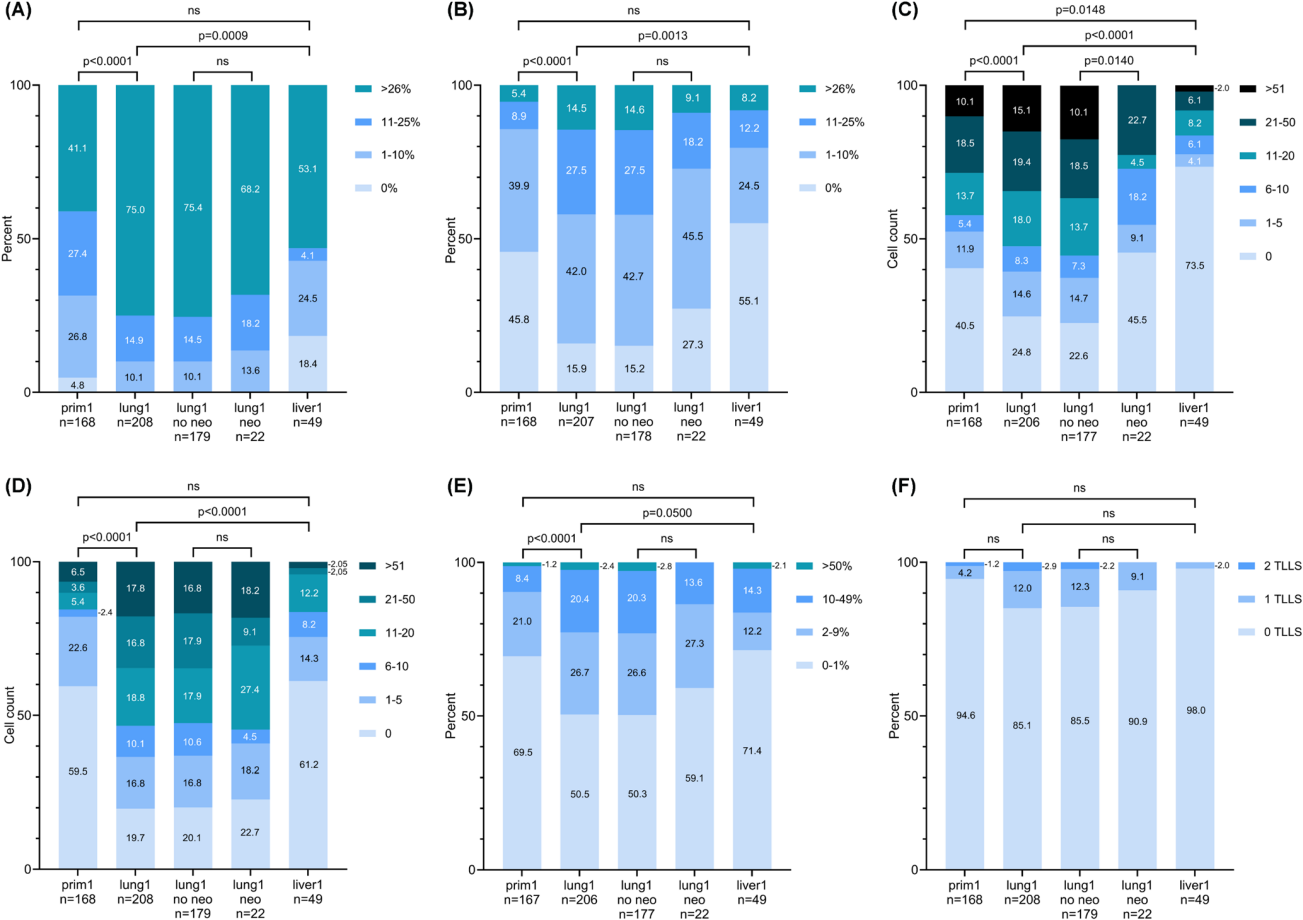
Distribution of immune marker expression across different tumor sites. Bar charts displaying the distribution of immune cell subsets (A) CD3, (B) CD8, (C) FoxP3, (D) CD20, (E) PD‐L1 on immune cells and (F) TLLS in primary tumors (prim1), initial/largest lung metastases (lung1), and initial/largest liver metastases (liver1), respectively. Lung metastases are stratified by neoadjuvant chemotherapy status (no neo vs. neo). Comparisons between primary tumors and metastases were performed using the Wilcoxon signed‐rank test; differences between lung subgroups were assessed using the Mann–Whitney *U* test. *p*‐values indicate statistical significance (ns = not significant).

Representative IHC images of immune markers in paired lung metastases and primary tumors are presented in Figure 1 and in Figure S1A. Bar charts visualizing the distribution of the investigated immune markers (Figure 2A–E) and the number of TLLSs (Figure 2F) across different locations, including before and after neoadjuvant chemotherapy in lung metastases, are presented in Figure 2. A significantly higher infiltration of all investigated immune cell subsets was observed in lung metastases compared to primary tumors (*p* < 0.0001) and liver metastases (*p* ≤ 0.05). The infiltration of CD3^+^, CD8^+^, CD20^+^, and PD‐L1^IC^ did not differ significantly between primary tumors and liver metastases, whereas the number of FoxP3^+^ cells was significantly lower in liver metastases compared to primary tumors (*p* = 0.0148). FoxP3^+^ cells co‐expressed CD3 (FoxP3^+^CD3^+^), and only occasionally FoxP3^+^CD3^−^ non‐T‐cells could be found (Figure S1A). Hence, we continued the analyses using FoxP3 as a marker for infiltrating FoxP3^+^ T‐cells. The number of PD‐L1^TC^ was overall low and did not differ between primary tumors and any metastatic site (Figure S1B).

The number of TLLS did not differ significantly between primary tumors and lung or liver metastases.

In lung metastases of patients who had not received neoadjuvant chemotherapy, the infiltration pattern of CD3^+^, CD8^+^, CD20^+^, and PD‐L1^IC^ and the number of TLLSs was similar compared to patients who had received neoadjuvant chemotherapy, except for the number of FoxP3^+^ cells that was significantly lower in the treated group (*p* = 0.0140) (Figure 2).

### 3.2 Clinicopathological Associations of Immune Cell Subsets and TLLSs

The distribution of patient and tumor characteristics in relation to categories of immune cell infiltration and TLLSs in primary tumors and lung metastases, respectively, is shown in Tables S1–S7. Overall, there were few significant correlations; high CD3^+^ in lung metastases correlated with a higher number of lung metastases, high CD8^+^ in lung metastases correlated with higher CRP before PM, high FoxP3^+^ in primary tumors correlated with lower CEA levels before surgery of the primary tumor, high CD20^+^ in primary tumors correlated with lower N stage, and TLLSs in the lung metastases correlated with a smaller size of the largest lung metastasis.

### 3.3 Prognostic Impact of Immune Markers in Lung Metastases and Corresponding Primary Tumors

Kaplan–Meier curves visualizing OS in the entire cohort according to all annotated strata of immune cell infiltration and the number of TLLSs in lung metastases are shown in Figure S2, and corresponding Kaplan–Meier curves stratified by adjuvant chemotherapy are shown in Figures S3 and S4. Univariable and multivariable Cox proportional hazards for death according to dichotomized immune marker variables are shown in Table S2. For CD3, FoxP3, CD20, and PD‐L1^IC^, dichotomous variables were applied based on visual inspection of Kaplan–Meier curves. For the number of TLLSs, dichotomous variables were applied based on their presence or absence. For CD8, where no evident differences were observed across potential cutoffs, the median was selected. Apart from a borderline significant association between high infiltration of CD3^+^ T cells and a longer OS in univariable analysis (*p* = 0.050), there were no significant associations between any of the other immune cell subsets or the number of TLLSs with OS (Table S8).

**FIGURE 3:**
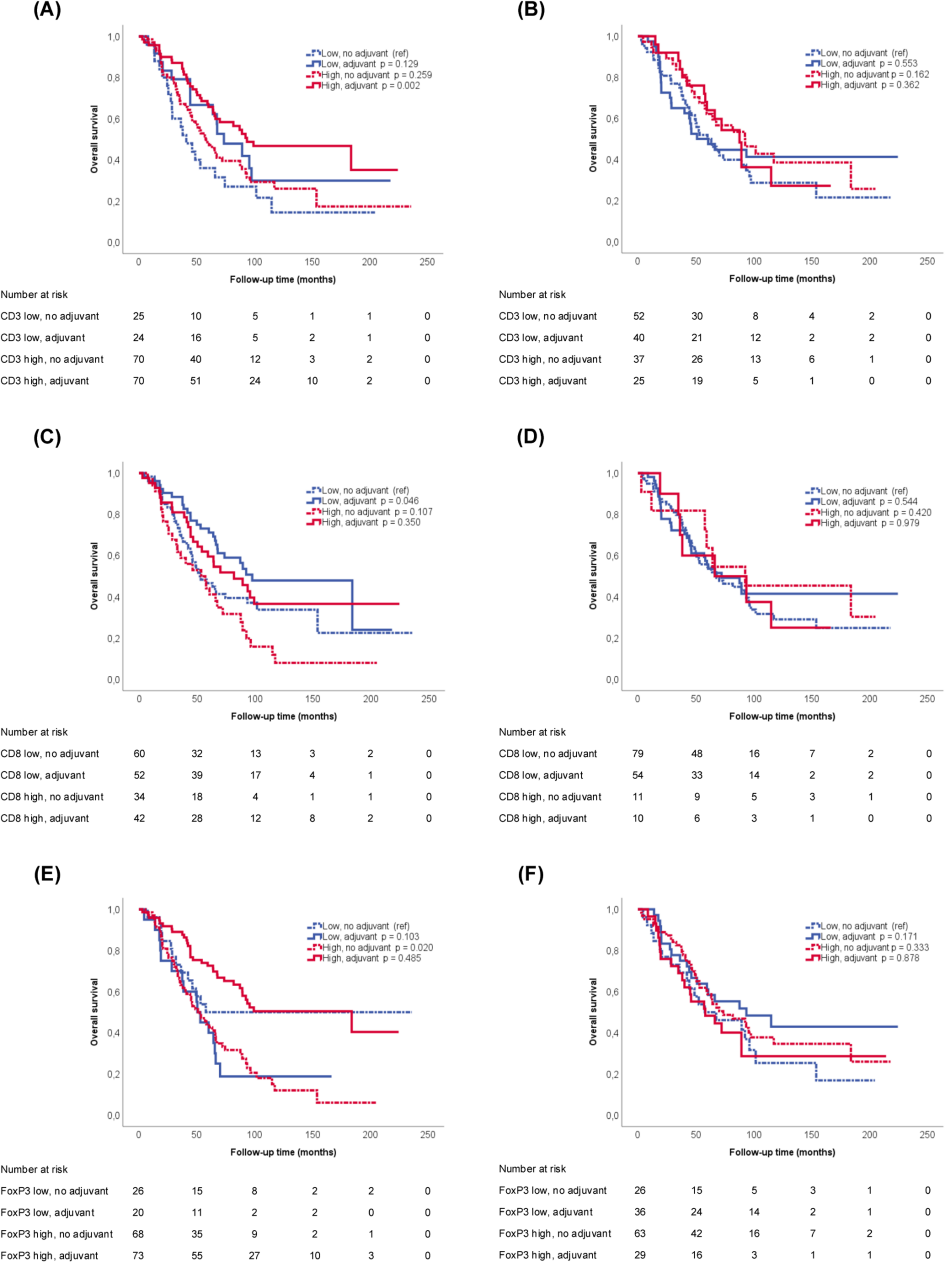
Kaplan–Meier graphs illustrating OS following PM, stratified by immune marker expression and adjuvant chemotherapy status. Survival according to treatment and CD3 expression in (A) lung metastases and (B) primary tumors, CD8 expression in (C) lung metastases and (D) primary tumors, and FoxP3 expression in (E) lung metastases and (F) primary tumors. The low expression/untreated group served as the reference category, and *p*‐values were calculated using the log‐rank test.

**FIGURE 4:**
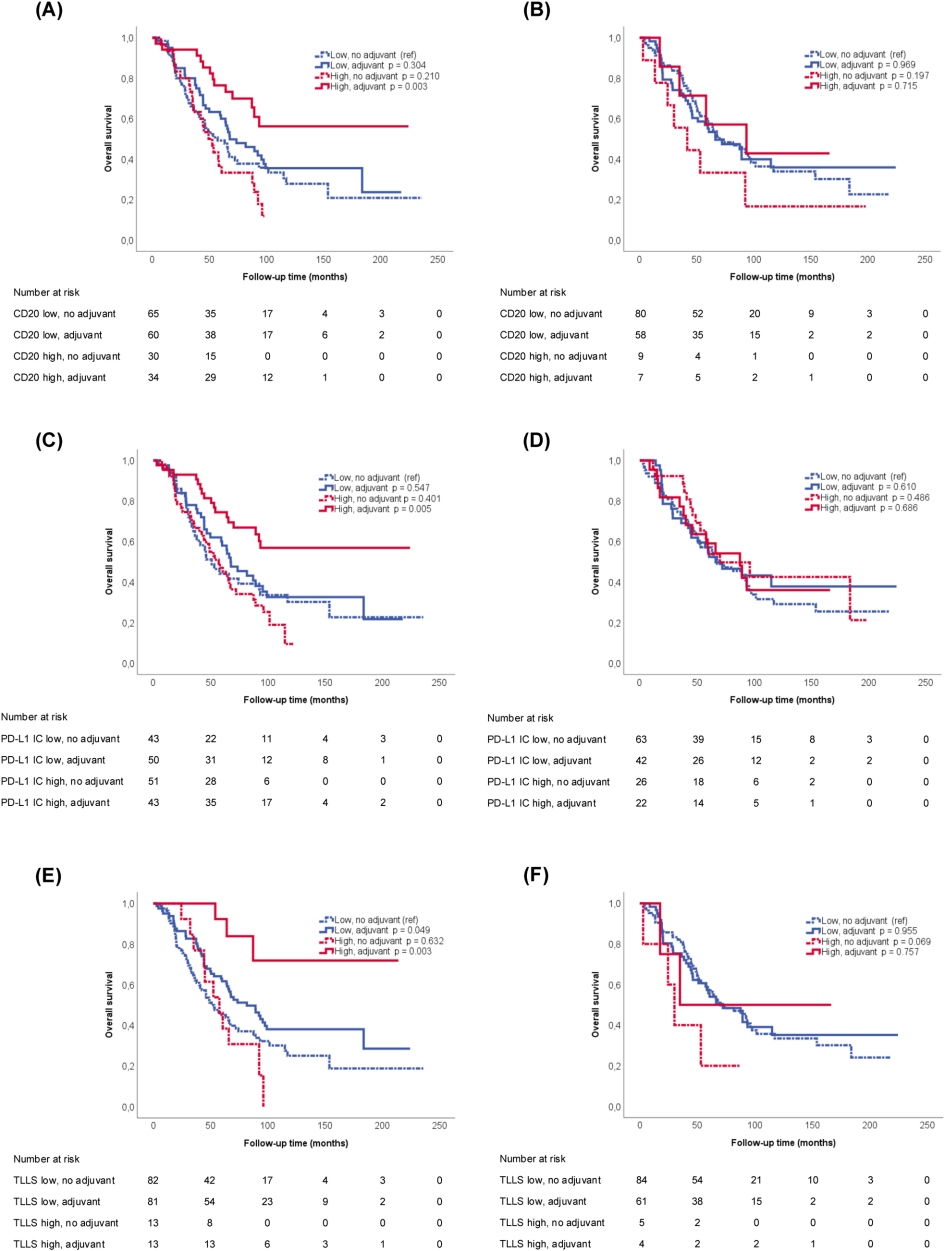
Kaplan–Meier curves illustrating OS following PM, stratified by immune marker expression and adjuvant chemotherapy status. Survival according to treatment and CD20 expression in (A) lung metastases and (B) primary tumors, PD‐L1 expression on immune cells in (C) lung metastases and (D) primary tumors, and the presence of TLLSs in (E) lung metastases and (F) primary tumors. The low expression/untreated group served as the reference category, and *p*‐values were calculated using the log‐rank test.

Next, we examined the prognostic value of high and low immune cell infiltration in lung metastases in relation to adjuvant chemotherapy. Corresponding Kaplan–Meier curves are shown in Figures 3 and 4. For patients treated with adjuvant chemotherapy, a prolonged OS was found when lung metastases had high infiltration of CD3^+^, CD20^+^, FoxP3^+^, and PD‐L1^IC^, or any number of TLLSs (Figure 3A and Figure 4A,C,E). This was not seen when immune infiltrates of the primary tumor were evaluated (Figure 3B and Figure 4B,D,F). In contrast, OS was longer in patients treated with adjuvant chemotherapy who had low compared to high infiltration of CD8+ in lung metastases, but not in primary tumors (Figure 3C,D). Of note, the strongest effect by adjuvant chemotherapy was found in patients with a high infiltration of FoxP3^+^ cells in lung metastases (Figure 3E and Table 1). Here, a high infiltration of FoxP3^+^ cells was significantly associated with a longer OS in adjuvant‐treated patients (*p <* 0.001; Table 1). As further shown in Table 1, Cox proportional hazards regression analysis with an interaction term confirmed a significant interaction between adjuvant chemotherapy and FoxP3^+^ immune cell infiltration in unadjusted analysis, which remained significant in adjusted analysis.

**TABLE 1:** Cox proportional hazards regression analysis of OS after PM, stratified by immune marker expression in lung metastases and adjuvant chemotherapy status.

	HR (95% CI)	*N*	*p*	*p*‐value for term of interaction, unadjusted analysis	*p*‐value for term of interaction, adjusted analysis*
***CD3 low***					
untreated	1.00	25			
treated	0.60 (0.31–1.17)	24	0.133		
***CD3 high***					
untreated	1.00	70			
treated	0.58 (0.38–0.89)	70	0.013	0.988	0.186
***CD8 low***					
untreated	1.00	60			
treated	0.61 (0.37–0.96)	52	0.048		
***CD8 high***					
untreated	1.00	34			
treated	0.52 (0.30–0.89)	42	0.018	0.684	0.763
***FoxP3 low***					
untreated	1.00	26			
treated	1.83 (0.88–3.80)	20	0.108		
***FoxP3 high***					
untreated	**1.00**	**68**			
treated	**0.37 (0.24–0.57)**	**73**	**< 0.001**	**< 0.001**	**0.001**
***CD20 low***					
untreated	1.00	65			
treated	0.80 (0.52–1.23)	60	0.305		
***CD20 high***					
untreated	1.00	30			
treated	0.30 (0.15–0.59)	34	< 0.001	0.016	0.473
***PD‐L1 low***					
untreated	1.00	43			
treated	0.86 (0.52–1.41)	50	0.548		
***PD‐L1 high***					
untreated	1.00	51			
treated	0.36 (0.20–0.63)	43	< 0.001	0.025	0.372
***TLLS low***					
untreated	1.00	82			
treated	0.68 (0.47–1.00)	81	0.05		
***TLLS high***					
untreated	1.00	13			
treated	0.14 (0.04–0.52)	13	0.003	0.042	0.886

Abbreviations: CI = confidence interval, HR = hazard ratio, OS = overall survival, PM = pulmonary metastasectomy, TLLS = tertiary lymphoid‐like structures.

* *p*‐value for term of interaction adjusted for age (continuous), > 1 metastasis, size of metastasis (continuous), DFI < 24 months vs. ≥ 24 months, N2 vs. N0‐N1, CEA > 5ug/L before PM, neoadjuvant vs. no neoadjuvant therapy before PM. HR and 95% CI are shown for each marker subgroup (low vs. high expression). Statistically significant values that remained significant in adjusted analysis are shown in boldS.

Kaplan–Meier curves visualizing OS in the entire cohort according to all annotated strata of immune cell infiltration and the number of TLLS in primary tumors are shown in Figure S5, corresponding Kaplan–Meier curves stratified by adjuvant chemotherapy are shown in Figures S6 and S7, and univariable and multivariable Cox proportional hazards analyses for death according to dichotomized variables using the same cutoffs as for lung metastases are shown in Table S9. No evident impact on OS was observed for any of the immune cell subsets or the number of TLLSs, and as further shown in Table 2, there was no significant interaction with adjuvant treatment.

**TABLE 2:** Cox proportional hazards regression analysis of overall survival after PM, stratified by immune marker expression in primary tumors and adjuvant chemotherapy status.

	HR (95% CI)	*N*	*p*	*p*‐value for term of interaction, unadjusted analysis	*p*‐value for term of interaction, adjusted analysis*
***CD3 low***					
untreated	1.00	52			
treated	0.85 (0.51–1.44)	40	0.553		
***CD3 high***					
untreated	1.00	37			
treated	1.15 (0.59–2.24)	25	0.684	0.345	0.599
***CD8 low***					
untreated	1.00	79			
treated	0.87 (0.56–1.36)	54	0.544		
***CD8 high***					
untreated	1.00	11			
treated	1.38 (0.46–4.11)	10	0.568	0.468	0.747
***FoxP3 low***					
untreated	1.00	26			
treated	0.64 (0.34–1.22)	36	0.174		
***FoxP3 high***					
untreated	1.00	63			
treated	1.31 (0.76–2.27)	29	0.337	0.089	0.098
***CD20 low***					
untreated	1.00	80			
treated	1.01 (0.65–1.56)	58	0.969		
***CD20 high***					
untreated	1.00	9			
treated	0.45 (0.13–1.58)	7	0.213	0.283	0.231
***PD‐L1 low***					
untreated	1.00	63			
treated	0.88 (0.53–1.45)	42	0.611		
***PD‐L1 high***					
untreated	1.00	26			
treated	1.14 (0.53–2.42)	22	0.744	0.628	0.812
***TLLS low***					
untreated	1.00	84			
treated	0.99 (0.65–1.51)	61	0.955		
***TLLS high***					
untreated	1.00	5			
treated	0.51 (0.92–2.78)	4	0.433	0.231	0.352

Abbreviations: CI = confidence interval, HR = hazard ratio, PM = pulmonary metastasectomy, TLLS = tertiary lymphoid‐like structures.

* *p*‐value for term of interaction adjusted for age (continuous), > 1 metastasis, size of metastasis (continuous), DFI < 24 months vs. ≥ 24 months, N2 vs. N0‐N1, CEA > 5ug/l before PM, neoadjuvant vs. no neoadjuvant therapy before PM. HR and 95% CI are shown for each marker subgroup (low vs. high expression). Statistically significant values are shown in bold.

Kaplan–Meier curves visualizing OS in the entire cohort according to all annotated strata of immune cell infiltration and the number of TLLSs in liver metastases are shown in Figure S8.

## 4 Discussion

This study aimed to characterize and compare the immune microenvironment of primary tumors and metastatic sites in mCRC, with focus on the prognostic and predictive value of tumor‐infiltrating immune cells. Although numerous studies have investigated tumor‐infiltrating immune cells and their prognostic relevance in mCRC, direct comparisons of the immune microenvironment between metastases and corresponding primary tumors remain scarce [14]. Our findings highlight a more immunologically active TME in lung metastases and suggest that immune infiltration may hold site‐specific predictive significance in the context of adjuvant chemotherapy. Understanding these differences is essential, as the immune contexture can influence treatment response and clinical outcomes. To our knowledge, this is among the largest studies that specifically assess the prognostic role of immune cell infiltration in resected colorectal lung metastases, in parallel with paired primary tumors.

We found significantly higher infiltration of all investigated immune cell subsets (CD3^+^, CD8^+^, CD20^+^, FoxP3^+^, and PD‐L1^IC^) in lung metastases compared to both primary tumors and liver metastases. This is consistent with previous studies [14, 18, 19, 20], and reinforces the notion that the lung and liver, the most common metastatic sites of CRC, harbor distinct immune environments. The lung is known for its immunologically active milieu [21], whereas the liver is characterized by an immunosuppressive environment [22], which may partly explain site‐specific responses to immunotherapy [23]. A higher PD‐L1^IC^ [14] and higher CD8^+^ T cell infiltration [18] have also been reported in lung metastases compared to primary tumors. Our findings support the observation of increased immune infiltration and PD‐L1^IC^ expression in lung metastases relative to both primary tumors and liver metastases. PD‐L1^TC^ expression was overall low and did not differ between primary tumors and any metastatic site, consistent with previous reports suggesting that PD‐L1^TC^ expression bears limited clinical significance in CRC [12]. However, not all findings are consistent across the literature. Schweiger et al. reported similar FoxP3^+^ T cell densities in paired lung metastases and primary tumors [20], while other studies have described higher CD8^+^ T cell infiltration in primary tumors than in lung metastases [24], or higher T cell densities in liver metastases relative to primary tumors [14, 25] and lung metastases [26], however, in the latter study, the differences were limited to the infiltrative margin and distant stroma, and not observed in the tumor region [26].

In the current study, CD3^+^, CD8^+^, CD20^+^, and PD‐L1^+^ immune cell infiltration did not differ significantly between primary tumors and liver metastases. This is in contrast to previous studies reporting both higher [14, 25] and lower immune cell infiltration in liver metastases [18, 27]. Importantly, however, we showed that FoxP3^+^ cells were significantly reduced in liver metastases. This aligns with a previous study demonstrating lower densities of FoxP3^+^ immune cells in liver metastases compared to paired primary tumors [28]. Although FoxP3^+^ cells are typically associated with immunosuppression, their functional heterogeneity [29] implies that reduced numbers do not necessarily reflect a less immunosuppressive microenvironment. The inherently tolerogenic immune milieu of the liver, involving alternative tumor‐promoting mechanisms such as myeloderived suppressor cells [30] and tumor‐associated macrophages [31], may play a more prominent role in immune evasion at this metastatic site. Further studies are warranted to elucidate the functional consequences of these findings.

TLLSs are key components of the adaptive immune response and have been associated with improved patient outcomes in several cancer types [32], as well as enhanced responses to immunotherapy [33]. In our cohort, the number of TLLSs did not differ significantly between primary tumors and lung or liver metastases. This is consistent with previous reports that also observed no significant differences in TLS density between lung metastases and primary tumors [16]. The lack of variation in TLLS density across metastatic sites may suggest that their presence is maintained during metastatic progression. However, further studies are needed to determine whether TLLS function or spatial organization is altered in the metastatic setting.

The infiltration of CD3^+^, CD8^+^, CD20^+^, and PD‐L1^IC +^ immune cells, as well as the number of TLLSs in lung metastases, did not differ significantly with neoadjuvant chemotherapy, which is in line with one previous study [26]. In contrast, the number of FoxP3^+^ cells was significantly lower in the treated group. FoxP3^+^ regulatory T cells (Tregs) are known to promote lung metastasis in CRC, partly via IL‐10‐mediated suppression of antitumor immunity [34]. Conventional chemotherapeutics can reduce Tregs [35, 36], and neoadjuvant chemotherapy has been shown to decrease Treg density in several cancers and increase CD8^+^ T cell infiltration in colorectal liver metastases [37].

When evaluating the prognostic relevance of individual immune cell subsets, high CD3^+^ T cell infiltration in lung metastases was only borderline significantly associated with longer OS in univariable analysis. No significant associations were observed for other markers or TLLSs. However, when stratifying the analysis according to adjuvant chemotherapy, patients with high infiltration of FoxP3^+^, CD20^+^, and PD‐L1^IC^, as well as those with any number of TLLSs in their lung metastases, had a significantly prolonged OS. In the stratified analyses, CD3 and CD8 only showed weak prognostic impact in patients treated with adjuvant chemotherapy. This is partly in line with a population‐based study of non‐resectable mCRC patients, where CD3^+^ and CD8^+^ cell infiltration in predominantly primary tumors did not show prognostic significance in the untreated group, but higher CD3^+^ T cell infiltration was associated with improved OS in patients who received palliative chemotherapy [38]. TLSs in CRC are generally associated with either favorable or neutral prognostic impact [17]. We found an association between their number and the efficacy of adjuvant treatment in lung metastases, where patients with a higher number of TLLSs experienced a more favorable treatment response, further confirmed in the unadjusted interaction model, although it did not remain significant after adjustment for clinicopathological factors. This is in contrast with a previous study that reported no prognostic value of TLS density in lung metastases [16], but aligns with the findings of another study, where the presence of TLSs in lung metastases was associated with favorable prognosis [24].

Our findings revealed a clear association between response to adjuvant chemotherapy and infiltrating FoxP3^+^ cells. Patients receiving adjuvant chemotherapy had an improved survival when lung metastases exhibited high infiltration of FoxP3^+^ cells. A Cox regression analysis with an interaction term confirmed a significant interaction between adjuvant chemotherapy and all immune markers as well as the number of TLLSs, with the association for FoxP3^+^ cells remaining significant in adjusted analysis. The vast majority of FoxP3^+^ cells also expressed the T‐cell marker CD3 (FoxP3^+^CD3^+^) although with varying intensity. While FoxP3^+^CD3^+^ cells are typically interpreted as Tregs, FoxP3 expression has also been reported in recently activated T‐cells, and in rare controversial cases even in non‐T‐cells [39, 40, 41, 42]. Our evaluation of FoxP3^+^ and CD3^+^ shows that the absolute majority of FoxP3 expressing cells in the present cohort are indeed CD3^+^ T‐cells. However, whether these are *bona fide* Tregs or recently activated T‐cells should be discussed with caution as activation markers like ICOS may also be expressed by Tregs [43, 44, 45]. The role of Tregs in CRC lung metastases is also complex. In general, Tregs suppress anti‐tumor immune responses within the TME [46], contributing to immune evasion by inhibiting effector T cell functions [37]. Expansion of the Treg population has been linked to increased cancer susceptibility and poorer clinical outcomes [47, 48], and meta‐analyses report associations between Treg infiltration and poor prognosis in several solid tumors [49, 50]. However, in CRC, Treg presence has also been associated with favorable outcomes [46, 49, 51], particularly in non‐metastatic disease up to stage III [52]. These divergent findings likely reflect the phenotypic and functional heterogeneity of intratumoural Tregs. Not all FoxP3^+^ Tregs exhibit equally potent immunosuppressive effects, and their potentially beneficial role may depend on the presence of chronic inflammation, as observed in CRC, distant ovarian cancer metastases, triple‐negative breast cancer, and smoking‐related esophageal squamous cell carcinoma [36]. In mCRC, higher FoxP3^+^ cell density has been linked to improved outcomes [53], while in colorectal liver metastases, Tregs appear to promote a more immunosuppressive environment and increased tumor aggressiveness [54]. This underscores the complexity of Treg biology and the need for caution in developing therapeutic strategies targeting Tregs in mCRC. In the context of colorectal lung metastases, the role of Tregs remains insufficiently understood. Lung‐resident Tregs may promote immunosuppression in the lung microenvironment, creating a permissive niche for metastatic colonization [9] and potentially limiting the efficacy of systemic therapies. Adjuvant chemotherapy may specifically target Tregs, which could explain the improved prognosis observed only in treated patients with lung metastases exhibiting high FoxP3^+^ cell infiltration [55]. Strategies targeting Treg function or IL‐10 production may hold therapeutic potential for limiting distant metastases in CRC [34]. Further research is warranted to clarify how Treg‐targeting approaches could enhance treatment responses in this setting.

From a clinical perspective, our findings indicate that immune profiling of resected lung metastases may help identify patients with colorectal lung metastases who are more likely to benefit from adjuvant chemotherapy. If validated, immune markers such as FoxP3^+^ T cells, CD20^+^ B cells, and PD‐L1^+^ immune cells could serve as predictive biomarkers in the metastatic setting, guiding personalized treatment strategies for these patients. As this is a retrospective study, there is an inherent risk of selection bias. Nevertheless, the analyzed tumors were clinically and histopathologically well‐characterized cases of mCRC. Other limitations include the single‐institution setting, the heterogeneity of treatment regimens, and relatively small subgroup sizes, all of which necessitate cautious interpretation, particularly of the predictive findings. While manual immune cell scoring may introduce some degree of variability, it reflects real‐world diagnostic practice and remains relevant, particularly in settings where automated image analysis tools are not yet validated for all markers. The dichotomization of immune cell densities into high and low categories may also introduce bias, especially in the absence of standardized thresholds [38, 56]. Various studies employ different approaches, such as median splits or the maximal chi‐square method, and the development of prospective, multicentre protocols is needed to define clinically relevant cutoffs [38, 57]. Another methodological consideration is the use of the TMA technique. While widely used in translational research, TMAs may not fully capture the heterogeneity of immune cell infiltration. Although sampling two 1 mm cores is generally regarded as adequate, our TMA design did not specifically target tumor stroma, which could influence results given the spatial variability of immune infiltration. Future studies should consider incorporating cores from both tumor and stromal compartments to provide a more comprehensive assessment of the tumor immune microenvironment. Moreover, spatially resolved approaches may offer further insight into the functional and prognostic relevance of immune cell localization within metastatic lesions. Together, our findings underscore the heterogeneity of the immune microenvironment in mCRC. While our results confirm increased immune infiltration in lung metastases, they contrast with previous studies and highlight the need for further investigation into the immunological landscape of mCRC. A more comprehensive understanding of these immune variations is essential to clarify their role in treatment response, particularly in the context of immunotherapy.

## 5 Conclusion

This study provides novel insight into the distinct immune microenvironments of primary tumors and metastases in mCRC, highlighting differences in immune cell infiltration and their prognostic interaction with adjuvant chemotherapy after pulmonary metastasectomy. The observed interaction between immune markers in lung metastases and adjuvant chemotherapy after PM underscores the importance of considering the immune landscape in lung metastases rather than in primary tumors when optimizing treatment strategies. Prospective studies are warranted to validate these findings and to elucidate the mechanisms underlying immune modulation by chemotherapy. A deeper understanding of immune heterogeneity across metastatic sites could ultimately guide more personalized therapeutic approaches in mCRC.

## Author Contributions

Danyil Kuznyecov: Formal analysis and writing of the original draft. Christina Siesing and Halla Vidarsdottir: Compilation of clinical data, review and editing of the original draft. Hans Brunnström: Histopathological re‐evaluation, cohort design, review and editing of the original draft. Jakob Eberhard: Supervision, review and editing of the original draft. Karin Leandersson: Supervision, review and editing of the original draft. Karin Jirström: Supervision, conceptualisation, methodology, resources, funding acquisition, review and editing of the original draft. All authors read and approved the final manuscript.

## Funding

This study was supported by grants from the Swedish Cancer Society (21‐1596 Pj, 24‐3644 Pj, CAN 2018‐418), the Mrs. Berta Kamprad Foundation (FBKS 2018‐15), Skåne Universital Hospital Donations and Funds, and Swedish governmental funding of clinical research (ALF).

## Ethics Statement

The study was approved by the regional ethical review board in Lund, Sweden (Dnr 2007/445, 2008/35, and 2014/748), whereby the committee waived the need for consent other than by the option to opt out.

## Conflicts of Interest

The authors declare no conflicts of interest.

## Supporting information


**Data S1:** Supplementary Tables.

**Data S2:** Supplementary Figures.

## Data Availability

The data that support the findings of this study are available on request from the corresponding author. The data are not publicly available due to privacy or ethical restrictions.
