# Supplementary material for: Tumor‐Infiltrating Immune Cells Are More Abundant in Lung Metastases From Colorectal Cancer Than in Paired Primary Tumors and Their Prognostic Value Depends on Adjuvant Chemotherapy
Source: Cancer Med. 2026 Mar 20;15(3):e71739. doi: 10.1002/cam4.71739 (PMC13093549; doi:10.1002/cam4.71739)
Supplement: Supplementary file 1 — Data S1: Supplementary Tables. [file CAM4-15-e71739-s002.docx]

SUPPLEMENTARY TABLES

# Supplementary Table 1. Correlations between CD3^+^ immune cells in lung metastases and primary tumours, respectively, and clinicopathological factors.

| **CD3** | **Primary tumour** | | | | **Lung metastases** | | | |
| --- | --- | --- | --- | --- | --- | --- | --- | --- |
| **Factor** | **N** | **Low** | **High** | **p-value*** | **N** | **Low** | **High** | **p-value*** |
| *Age at first pulmonary metastasectomy* | 168 |  |  |  | 208 |  |  |  |
| ≤60 years | 39 | 26 (66.7) | 13 (33.3) | 0.262 | 48 | 10 (20.8) | 38 (79.2) | 0.447 |
| >60 years | 129 | 73 (56.6) | 56 (43.4) |  | 160 | 42 (26.3) | 118 (73.8) |  |
| *Gender* | 168 |  |  |  | 208 |  |  |  |
| Male | 97 | 55 (56.7) | 42 (43.3) | 0.493 | 123 | 31 (25.2) | 92 (74.8) | 0.935 |
| Female | 71 | 44 (62.0) | 27 (38.0) |  | 85 | 21 (24.7) | 64 (75.3) |  |
| *Synchronous metastasis* | 168 |  |  |  | 208 |  |  |  |
| Yes | 39 | 19 (48.7) | 20 (51.3) | 0.139 | 50 | 12 (24.0) | 38 (76.0) | 0.851 |
| No | 129 | 80 (62.0) | 49 (38.0) |  | 158 | 40 (25.3) | 118 (74.7) |  |
| *Disease-free interval* | 168 |  |  |  | 208 |  |  |  |
| ≤24 months | 102 | 60 (58.8) | 42 (41.2) | 0.973 | 121 | 30 (24.8) | 91 (75.2) | 0.935 |
| >24 months | 66 | 39 (59.1) | 27 (40.9) |  | 87 | 22 (25.3) | 65 (74.7) |  |
| *CEA before metastasectomy* | 105 |  |  |  | 125 |  |  |  |
| ≤5 μg /L | 83 | 50 (60.2) | 33 (39.8) | 0.772 | 95 | 26 (10.5) | 69 (89.5) | 0.529 |
| >5 μg /L | 22 | 14 (63.6) | 8 (36.4) |  | 30 | 10 (33.3) | 20 (66.7) |  |
| *CRP before metastasectomy* | 149 |  |  |  | 182 |  |  |  |
| ≤10 mg/L | 135 | 81 (60.0) | 54 (40.0) | 0.469 | 164 | 39 (23.8) | 125 (76.2) | 0.707 |
| >10 mg/L | 14 | 7 (50.0) | 7 (50.0) |  | 18 | 5 (27.8) | 13 (72.2) |  |
| *Location of primary tumour* | 168 |  |  |  | 208 |  |  |  |
| Right colon | 16 | 10 (62.5) | 6 (37.5) | 0.737 | 16 | 7 (43.8) | 9 (56.3) | 0.192 |
| Left colon | 60 | 33 (55.0) | 27 (45.0) |  | 71 | 16 (22.5) | 55 (77.5) |  |
| Rectum | 92 | 56 (60.9) | 36 (39.1) |  | 121 | 29 (24.0) | 92 (76.0) |  |
| *Primary tumour stage* |  |  |  |  |  |  |  |  |
| T stage | 160 |  |  |  | 189 |  |  |  |
| 1 or 2 | 24 | 11 (45.8) | 13 (54.2) | 0.368 | 32 | 9 (28.1) | 23 (71.9) | 0.891 |
| 3 | 110 | 67 (60.9) | 43 (39.1) |  | 129 | 31 (24.0) | 98 (76.0) |  |
| 4 | 26 | 14 (53.8) | 12 (46.2) |  | 28 | 7 (25.0) | 21 (75.0) |  |
| N stage | 163 |  |  |  | 196 |  |  |  |
| 0 | 60 | 32 (53.3) | 28 (46.7) | 0.517 | 81 | 23 (28.4) | 58 (71.6) | 0.559 |

| 1 | 63 | 40 (63.5) | 23 (36.5) |  | 72 | 16 (22.2) | 56 (77.8) |  |
| --- | --- | --- | --- | --- | --- | --- | --- | --- |
| 2 | 40 | 23 (57.5) | 17 (42.5) |  | 43 | 9 (20.9) | 34 (79.1) |  |
| *CEA before surgery of primary tumour* | 83 |  |  |  | 93 |  |  |  |
| ≤5 ng/mL | 51 | 31 (60.8) | 20 (39.2) | 0.898 | 55 | 12 (21.8) | 43 (78.2) | 0.433 |
| >5 ng/mL | 32 | 19 (59.4) | 13 (40.6) |  | 38 | 11 (28.9) | 27 (71.1) |  |
| *Surgery for liver metastasis* | 168 |  |  |  | 208 |  |  |  |
| Yes | 58 | 34 (58.6) | 24 (41.4) | 0.953 | 66 | 18 (27.3) | 48 (72.7) | 0.606 |
| No | 110 | 65 (59.1) | 45 (40.9) |  | 142 | 34 (23.9) | 108 (76.1) |  |
| *Number of lung metastases* | 168 |  |  |  | 208 |  |  |  |
| 1 | 117 | 67 (57.3) | 50 (42.7) | 0.507 | **144** | **29 (20.1)** | **115 (79.9)** | **0.015** |
| ≥2 | 51 | 32 (62.7) | 19 (37.3) |  | **64** | **23 (35.9)** | **41 (64.1)** |  |
| *Size of lung metastases* | 158 |  |  |  | 198 |  |  |  |
| ≤3 cm | 136 | 75 (55.1) | 61 (44.9) | 0.252 | 167 | 38 (22.8) | 129 (77.2) | 0.257 |
| >3 cm | 22 | 15 (68.2) | 7 (31.8) |  | 31 | 10 (32.3) | 21 (67.7) |  |

*P-values were calculated using the Chi-square test. Low: <25% positive cells. High: ≥25% positive cells. CEA=carcinoembryonic antigen, CRP=C‐reactive protein.

# Supplementary Table 2. Correlations between CD8^+^ immune cells in lung metastases and primary tumours, respectively, and clinicopathological factors.

| **CD8** | **Primary tumour** | | | | **Lung metastases** | | | |
| --- | --- | --- | --- | --- | --- | --- | --- | --- |
| **Factor** | **N** | **Low** | **High** | **p-value*** | **N available** | **Low** | **High** | **p-value*** |
| *Age at first pulmonary metastasectomy* | 168 |  |  |  | 207 |  |  |  |
| ≤60 years | 38 | 36 (94.7) | 2 (5.3) | 0.071 | 48 | 27 (56.3) | 21 (43.8) | 0.783 |
| >60 years | 130 | 108 (83.1) | 22 (16.9) |  | 159 | 93 (58.5) | 66 (41.5) |  |
| *Gender* | 168 |  |  |  | 207 |  |  |  |
| Male | 97 | 83 (85.6) | 14 (14.4) | 0.949 | 122 | 77 (63.1) | 45 (36.9) | 0.072 |
| Female | 71 | 61 (85.9) | 10 (14.1) |  | 85 | 43 (50.6) | 42 (49.4) |  |
| *Synchronous metastasis* | 168 |  |  |  | 207 |  |  |  |
| Yes | 40 | 34 (85.0) | 6 (15.0) | 0.882 | 50 | 24 (48.0) | 26 (52.0) | 0.101 |
| No | 128 | 110 (85.9) | 18 (14.1) |  | 157 | 96 (61.1) | 61 (38.9) |  |
| *Disease-free interval* | 168 |  |  |  | 207 |  |  |  |
| ≤24 months | 102 | 89 (87.3) | 13 (12.7) | 0.478 | 121 | 69 (57.0) | 52 (43.0) | 0.744 |
| >24 months | 66 | 55 (83.3) | 11 (16.7) |  | 86 | 51 (59.3) | 35 (40.7) |  |
| *CEA before metastasectomy* | 105 |  |  |  | 125 |  |  |  |
| ≤5 μg /L | 82 | 74 (90.2) | 8 (9.8) | 0.649 | 95 | 63 (66.3) | 32 (33.7) | 0.108 |
| >5 μg /L | 23 | 20 (87.0) | 3 (13.0) |  | 30 | 15 (50.0) | 15 (50.0) |  |
| *CRP before metastasectomy* | 148 |  |  |  | 181 |  |  |  |
| ≤10 mg/L | 134 | 118 (88.1) | 16 (11.9) | 0.313 | **163** | **103 (63.2)** | **60 (36.8)** | **0.042** |
| >10 mg/L | 14 | 11 (78.6) | 3 (21.4) |  | **18** | **7 (38.9)** | **11 (61.1)** |  |
| *Location of primary tumour* | 168 |  |  |  | 207 |  |  |  |
| Right colon | 16 | 13 (81.25) | 3 (18.75) | 0.623 | 16 | 12 (75.0) | 4 (25.0) | 0.356 |
| Left colon | 60 | 50 (83.3) | 10 (16.7) |  | 71 | 40 (56.3) | 31 (43.7) |  |
| Rectum | 92 | 81 (47.7) | 11 (52.3) |  | 120 | 58 (56.7) | 62 (43.3) |  |
| *Primary tumour stage* |  |  |  |  |  |  |  |  |
| T stage | 160 |  |  |  | 188 |  |  |  |
| 1 or 2 | 24 | 21 (87.5) | 3 (12.5) | 0.438 | 32 | 17 (53.1) | 15 (46.9) | 0.337 |
| 3 | 110 | 91 (82.7) | 19 (17.3) |  | 128 | 76 (59.4) | 52 (40.6) |  |
| 4 | 26 | 24 (92.3) | 2 (7.7) |  | 28 | 20 (71.4) | 8 (28.6) |  |
| N stage | 163 |  |  |  | 195 |  |  |  |
| 0 | 61 | 53 (86.9) | 8 (13.1) | 0.893 | 81 | 46 (56.8) | 35 (43.2) | 0.811 |

| 1 | 62 | 52 (83.9) | 10 (16.1) |  | 71 | 42 (59.2) | 29 (40.8) |  |
| --- | --- | --- | --- | --- | --- | --- | --- | --- |
| 2 | 40 | 34 (85.0) | 6 (15.0) |  | 43 | 27 (62.8) | 16 (37.2) |  |
| *CEA before surgery of primary tumour* | 82 |  |  |  | 92 |  |  |  |
| ≤5 ng/mL | 50 | 19 (38.0) | 3 (62.0) | 0.426 | 55 | 37 (67.3) | 18 (32.7) | 0.444 |
| >5 ng/mL | 32 | 15 (46.99 | 17 (53.1) |  | 37 | 22 (59.5) | 15 (40.5) |  |
| *Surgery for liver metastasis* | 168 |  |  |  | 207 |  |  |  |
| Yes | 57 | 48 (84.2) | 9 (15.8) | 0.690 | 65 | 39 (60.0) | 26 (40.0) | 0.689 |
| No | 111 | 96 (86.5) | 15 (13.5) |  | 142 | 81 (57.0) | 61 (43.0) |  |
| *Number of lung metastases* | 168 |  |  |  | 207 |  |  |  |
| 1 | 118 | 100 (84.7) | 18 (15.3) | 0.582 | 143 | 83 (58.0) | 60 (42.0) | 0.975 |
| ≥2 | 40 | 44 (88.0) | 6 (12.0) |  | 64 | 37 (57.8) | 27 (42.2) |  |
| *Size of lung metastases* | 158 |  |  |  | 197 |  |  |  |
| ≤3 cm | 136 | 117 (86.0) | 19 (14.0) | 0.966 | 166 | 100 (60.2) | 66 (39.8) | 0.059 |
| >3 cm | 22 | 19 (86.4) | 3 (13.6) |  | 31 | 13 (41.9) | 18 (58.1) |  |

*P-values were calculated using the Chi-square test. Low: <10% positive cells. High: ≥10% positive cells. CEA=carcinoembryonic antigen, CRP=C‐reactive protein.

# Supplementary Table 3. Correlations between FoxP3^+^ immune cells in lung metastases and primary tumours, respectively, and clinicopathological factors.

| **FoxP3** | **Primary tumour** | | | | **Lung metastases** | | | |
| --- | --- | --- | --- | --- | --- | --- | --- | --- |
| **Factor** | **N** | **Low** | **High** | **p-value*** | **N** | **Low** | **High** | **p-value*** |
| *Age at first pulmonary metastasectomy* | 168 |  |  |  | 206 |  |  |  |
| ≤60 years | 39 | 13 (33.3) | 26 (66.7) | 0.300 | 48 | 14 (29.2) | 34 (70.8) | 0.419 |
| >60 years | 129 | 55 (42.6) | 74 (57.4) |  | 158 | 37 (23.4) | 121 (76.6) |  |
| *Gender* | 168 |  |  |  | 206 |  |  |  |
| Male | 97 | 41 (42.3) | 56 (57.7) | 0.580 | 122 | 34 (27.9) | 88 (72.1) | 0.212 |
| Female | 71 | 27 (38.0) | 44 (62.0) |  | 84 | 17 (20.2) | 67 (79.8) |  |
| *Synchronous metastasis* | 168 |  |  |  | 206 |  |  |  |
| Yes | 40 | 12 (30.0) | 28 (70.0) | 0.122 | 50 | 11 (22.0) | 39 (78.0) | 0.604 |
| No | 128 | 56 (43.8) | 72 (56.3) |  | 156 | 40 (25.6) | 116 (74.4) |  |
| *Disease-free interval* | 168 |  |  |  | 206 |  |  |  |
| ≤24 months | 103 | 36 (35.0) | 67 (65.0) | 0.066 | 120 | 29 (24.2) | 91 (75.8) | 0.817 |
| >24 months | 65 | 32 (49.2) | 33 (50.8) |  | 86 | 22 (25.6) | 64 (74.4) |  |
| *CEA before metastasectomy* | 105 |  |  |  | 124 |  |  |  |
| ≤5 μg /L | 83 | 35 (42.2) | 48 (57.8) | 0.510 | 94 | 22 (23.4) | 72 (76.6) | 0.076 |
| >5 μg /L | 22 | 11 (50.0) | 11 (50.0) |  | 30 | 12 (40.0) | 18 (60.0) |  |
| *CRP before metastasectomy* | 148 |  |  |  | 180 |  |  |  |
| ≤10 mg/L | 134 | 59 (44.0) | 75 (56.0) | 0.550 | 162 | 43 (26.5) | 119 (73.5) | 0.539 |
| >10 mg/L | 14 | 5 (35.7) | 9 (64.3) |  | 18 | 6 (33.3) | 12 (66.7) |  |
| *Location of primary tumour* | 168 |  |  |  | 206 |  |  |  |
| Right colon | 16 | 7 (43.75) | 9 (56.25) | 0.900 | 16 | 4 (25.0) | 12 (75.0) | 0.699 |
| Left colon | 60 | 23 (38.3) | 37 (61.7) |  | 71 | 20 (28.2) | 51 (71.8) |  |
| Rectum | 92 | 38 (41.3) | 54 (58.7) |  | 119 | 27 (22.7) | 92 (77.3) |  |
| *Primary tumour stage* |  |  |  |  |  |  |  |  |
| T stage | 160 |  |  |  | 187 |  |  |  |
| 1 or 2 | 24 | 7 (29.2) | 17 (70.8) | 0.323 | **32** | **5 (15.6)** | **27 (84.4)** | **0.041** |
| 3 | 110 | 45 (40.9) | 65 (59.1) |  | **127** | **30 (23.6)** | **97 (76.4)** |  |
| 4 | 26 | 13 (50.0) | 13 (50.0) |  | **28** | **12 (42.9)** | **16 (57.1)** |  |
| N stage | 163 |  |  |  | 194 |  |  |  |
| 0 | 60 | 24 (40.0) | 36 (60.0) | 0.987 | 81 | 16 (19.8) | 65 (80.2) | 0.164 |

| 1 | 63 | 26 (41.3) | 37 (58.7) |  | 70 | 16 (22.9) | 54 (77.1) |  |
| --- | --- | --- | --- | --- | --- | --- | --- | --- |
| 2 | 40 | 16 (40.0) | 24 (60.0) |  | 43 | 15 (34.9) | 28 (65.1) |  |
| *CEA before surgery of primary tumour* | 84 |  |  |  | 91 |  |  |  |
| ≤5 ng/mL | **51** | **17 (33.3)** | **34 (66.7)** | **0.014** | 54 | 12 (22.2) | 42 (77.8) | 0.815 |
| >5 ng/mL | **33** | **20 (60.6)** | **13 (39.4)** |  | 37 | 9 (24.3) | 28 (75.7) |  |
| *Surgery for liver metastasis* | 168 |  |  |  | 206 |  |  |  |
| Yes | 57 | 24 (42.1) | 33 (57.9) | 0.758 | 65 | 19 (29.2) | 46 (70.8) | 0.312 |
| No | 111 | 44 (39.6) | 67 (60.4) |  | 141 | 32 (22.7) | 109 (77.3) |  |
| *Number of lung metastases* | 168 |  |  |  | 206 |  |  |  |
| 1 | 117 | 48 (41.0) | 69 (59.0) | 0.826 | 143 | 33 (23.1) | 110 (76.9) | 0.400 |
| ≥2 | 51 | 20 (39.2) | 31 (60.8) |  | 63 | 18 (28.6) | 45 (71.4) |  |
| *Size of lung metastases* | 158 |  |  |  | 196 |  |  |  |
| ≤3 cm | 136 | 53 (39.0) | 83 (61.0) | 0.564 | 165 | 37 (22.4) | 128 (77.6) | 0.426 |
| >3 cm | 22 | 10 (45.5) | 12 (54.5) |  | 31 | 9 (29.0) | 22 (71.0) |  |

*P-values were calculated using the Chi-square test. Low: <1 positive cell. High: ≥1 positive cell. CEA=carcinoembryonic antigen, CRP=C‐reactive protein.

# Supplementary Table 4. Correlations between CD20^+^ immune cells in lung metastases and primary tumours, respectively, and clinicopathological factors.

| **CD20** | **Primary tumour** | | | | **Lung metastases** | | | |
| --- | --- | --- | --- | --- | --- | --- | --- | --- |
| **Factor** | **N** | **Low** | **High** | **p-value*** | **N available** | **Low** | **High** | **p-value*** |
| *Age at first pulmonary metastasectomy* | 168 |  |  |  | 208 |  |  |  |
| ≤60 years | 39 | 35 (89.7) | 4 (10.3) | 0.974 | 48 | 28 (58.3) | 20 (41.7) | 0.242 |
| >60 years | 129 | 116 (89.9) | 13 (10.1) |  | 160 | 108 (67.5) | 52 (32.5) |  |
| *Gender* | 168 |  |  |  | 208 |  |  |  |
| Male | 97 | 85 (87.6) | 12 (12.4) | 0.258 | 123 | 82 (66.7) | 41 (33.3) | 0.640 |
| Female | 71 | 66 (93.0) | 5 (7.0) |  | 85 | 54 (63.5) | 31 (36.5) |  |
| *Synchronous metastasis* | 168 |  |  |  | 208 |  |  |  |
| Yes | 39 | 35 (89.9) | 4 (10.1) | 0.974 | 50 | 30 (60.0) | 20 (40.0) | 0.358 |
| No | 129 | 116 (88.9) | 13 (10.1) |  | 158 | 106 (97.1) | 52 (32.9) |  |
| *Disease-free interval* | 168 |  |  |  | 211 |  |  |  |
| ≤24 months | 102 | 90 (88.2) | 12 (11.8) | 0.379 | 121 | 76 (62.8) | 45 (37.2) | 0.357 |
| >24 months | 66 | 61 (92.4) | 5 (7.6) |  | 87 | 60 (69.0) | 27 (31.0) |  |
| *CEA before metastasectomy* | 105 |  |  |  | 125 |  |  |  |
| ≤5 μg /L | 83 | 73 (88.0) | 10 (12.0) | 0.307 | 97 | 64 (67.4) | 31 (32.6) | 0.943 |
| >5 μg /L | 22 | 21 (95.5) | 1 (4.5) |  | 30 | 20 (66.7) | 10 (33.3) |  |
| *CRP before metastasectomy* | 149 |  |  |  | 182 |  |  |  |
| ≤10 mg/L | 135 | 122 (90.4) | 13 (9.6) | 0.175 | 164 | 102 (62.2) | 62 (37.8) | 0.192 |
| >10 mg/L | 14 | 11 (78.6) | 3 (21.4) |  | 18 | 14 (77.8) | 4 (22.2) |  |
| *Location of primary tumour* | 168 |  |  |  | 208 |  |  |  |
| Right colon | 16 | 16 (100.0) | 0 (0.0) | 0.342 | 16 | 14 (87.5) | 2 (12.5) | 0.123 |
| Left colon | 60 | 54 (90.0) | 6 (10.0) |  | 71 | 43 (60.6) | 28 (39.4) |  |
| Rectum | 92 | 81 (88.0) | 11 (12.0) |  | 121 | 79 (65.3) | 42 (34.7) |  |
| *Primary tumour stage* |  |  |  |  |  |  |  |  |
| T stage | 160 |  |  |  | 189 |  |  |  |
| 1 or 2 | 24 | 22 (91.7) | 2 (8.3) | 0.386 | 32 | 24 (75.0) | 8 (25.0) | 0.149 |
| 3 | 110 | 96 (87.3) | 14 (12.7) |  | 129 | 78 (60.5) | 51 (39.5) |  |
| 4 | 26 | 25 (96.2) | 1 (3.8) |  | 28 | 21 (75.0) | 7 (25.0) |  |
| N stage | 163 |  |  |  | 196 |  |  |  |
| 0 | 60 | **54 (90.0)** | **6 (10.0)** | **0.047** | 81 | 58 (71.6) | 23 (28.4) | 0.131 |

| 1 | 63 | **60 (95.2)** | **3 (4.8)** |  | 72 | 47 (65.3) | 25 (34.7) |  |
| --- | --- | --- | --- | --- | --- | --- | --- | --- |
| 2 | 40 | **32 (80.0)** | **8 (20.0)** |  | 43 | 23 (53.5) | 20 (46.5) |  |
| *CEA before surgery of primary tumour* | 83 |  |  |  | 93 |  |  |  |
| ≤5 ng/mL | 51 | 45 (88.2) | 6 (11.8) | 0.733 | 55 | 34 (61.8) | 21 (38.2) | 0.696 |
| >5 ng/mL | 32 | 29 (90.6) | 3 (9.4) |  | 38 | 25 (65.8) | 13 (34.2) |  |
| *Surgery for liver metastasis* | 168 |  |  |  | **208** |  |  |  |
| Yes | 58 | 51 (87.9) | 7 (12.1) | 0.543 | **66** | **50 (75.8)** | **16 (24.2)** | **0.032** |
| No | 110 | 100 (90.9) | 10 (9.1) |  | **142** | **86 (60.6)** | **56 (39.4)** |  |
| *Number of lung metastases* | 168 |  |  |  | 208 |  |  |  |
| 1 | 117 | 106 (90.6) | 11 (9.4) | 0.641 | 144 | 92 (63.9) | 52 (36.1) | 0.496 |
| ≥2 | 51 | 45 (88.2) | 6 (11.8) |  | 64 | 44 (68.8) | 20 (31.3) |  |
| *Size of lung metastases* | 158 |  |  |  | 198 |  |  |  |
| ≤3 cm | 136 | 120 (88.2) | 16 (11.8) | 0.090 | 167 | 103 (61.7) | 64 (38.3) | 0.093 |
| >3 cm | 22 | 22 (100.0) | 0 (0.0) |  | 31 | 24 (77.4) | 7 (22.6) |  |

*P-values were calculated using the Chi-square test. Low: <20 positive cells. High: ≥20 positive cells. CEA=carcinoembryonic antigen, CRP=C‐reactive protein.

# Supplementary Table 5. Correlations between PD-L1^+^ immune cells in lung metastases and primary tumours, respectively, and clinicopathological factors.

| **PD-L1^IC^** | **Primary tumour** | | | | **Lung metastases** | | | |
| --- | --- | --- | --- | --- | --- | --- | --- | --- |
| **Factor** | **N** | **Low** | **High** | **p-value*** | **N available** | **Low** | **High** | **p-value*** |
| *Age at first pulmonary metastasectomy* | 167 |  |  |  | 206 |  |  |  |
| ≤60 years | 38 | 29 (76.3) | 9 (23.7) | 0.297 | 48 | 25 (52.1) | 23 (47.9) | 0.800 |
| >60 years | 129 | 87 (67.4) | 42 (32.6) |  | 158 | 79 (50.0) | 79 (50.0) |  |
| *Gender* | 167 |  |  |  | 206 |  |  |  |
| Male | 97 | 68 (70.1) | 29 (29.9) | 0.832 | 122 | 65 (53.3) | 57 (46.7) | 0.334 |
| Female | 70 | 48 (68.6) | 22 (31.4) |  | 84 | 39 (46.4) | 45 (53.6) |  |
| *Synchronous metastasis* | 167 |  |  |  | 206 |  |  |  |
| Yes | 40 | 22 (55.0) | 18 (45.0) | 0.090 | 50 | 28 (56.0) | 22 (44.0) | 0.370 |
| No | 127 | 94 (74.0) | 33 (26.0) |  | 156 | 76 (48.7) | 80 (51.3) |  |
| *Disease-free interval* | 167 |  |  |  | 206 |  |  |  |
| ≤24 months | 103 | 68 (66.0) | 35 (34.0) | 0.221 | 120 | 65 (54.2) | 55 (45.8) | 0.212 |
| >24 months | 64 | 48 (75.0) | 16 (25.0) |  | 86 | 39 (45.3) | 47 (54.7) |  |
| *CEA before metastasectomy* | 105 |  |  |  | 124 |  |  |  |
| ≤5 μg /L | 83 | 58 (69.9) | 25 (30.1) | 0.495 | 94 | 43 (45.7) | 51 (54.3) | 0.297 |
| >5 μg /L | 22 | 17 (77.3) | 5 (22.7) |  | 30 | 17 (56.7) | 13 (43.3) |  |
| *CRP before metastasectomy* | 147 |  |  |  | 180 |  |  |  |
| ≤10 mg/L | 133 | 91 (68.4) | 42 (31.6) | 0.752 | 162 | 75 (46.3) | 87 (53.7) | 0.101 |
| >10 mg/L | 14 | 9 (64.3) | 5 (35.7) |  | 18 | 12 (66.7) | 6 (33.3) |  |
| *Location of primary tumour* | 167 |  |  |  | 206 |  |  |  |
| Right colon | 15 | 11 (73.3) | 4 (26.7) | 0.931 | 16 | 11 (68.75) | 5 (31.25) | 0.224 |
| Left colon | 60 | 41 (68.3) | 19 (31.7) |  | 71 | 32 (45.1) | 39 (54.9) |  |
| Rectum | 92 | 64 (69.6) | 28 (30.4) |  | 119 | 61 (51.3) | 58 (48.7) |  |
| *Primary tumour stage* |  |  |  |  |  |  |  |  |
| T stage | **159** |  |  |  | 187 |  |  |  |
| 1 or 2 | **22** | **19 (79.2)** | **5 (20.8)** | **0.044** | 32 | 18 (56.3) | 14 (43.8) | 0.869 |
| 3 | **110** | **68 (61.8)** | **42 (38.2)** |  | 127 | 65 (51.2) | 62 (48.8) |  |
| 4 | **25** | **21 (84.0)** | **4 (16.0)** |  | 28 | 15 (53.6) | 13 (46.4) |  |
| N stage | 162 |  |  |  | 194 |  |  |  |
| 0 | 60 | 41 (68.3) | 19 (31.7) | 0.564 | 81 | 42 (51.9) | 39 (48.1) | 0.792 |

| 1 | 62 | 45 (72.6) | 17 (27.4) |  | 70 | 37 (52.9) | 33 (47.1) |  |
| --- | --- | --- | --- | --- | --- | --- | --- | --- |
| 2 | 40 | 25 (62.5) | 15 (37.5) |  | 43 | 20 (46.5) | 23 (53.5) |  |
| *CEA before surgery of primary tumour* | 84 |  |  |  | 91 |  |  |  |
| ≤5 ng/mL | 51 | 33 (64.7) | 18 (35.3) | 0.442 | 54 | 26 (48.1) | 28 (51.9) | 0.645 |
| >5 ng/mL | 33 | 24 (72.7) | 9 (27.3) |  | 37 | 16 (43.2) | 21 (56.8) |  |
| *Surgery for liver metastasis* | 167 |  |  |  | 206 |  |  |  |
| Yes | 57 | 39 (68.4) | 18 (31.6) | 0.834 | 65 | 32 (49.2) | 33 (50.8) | 0.807 |
| No | 110 | 77 (70.0) | 33 (30.0) |  | 141 | 72 (51.1) | 69 (48.9) |  |
| *Number of lung metastases* | 167 |  |  |  | 206 |  |  |  |
| 1 | 116 | 76 (65.5) | 40 (34.5) | 0.095 | 143 | 67 (46.9) | 76 (53.1) | 0.116 |
| ≥2 | 51 | 40 (78.4) | 11 (21.6) |  | 63 | 37 (58.7) | 26 (41.3) |  |
| *Size of lung metastases* | 155 |  |  |  | 196 |  |  |  |
| ≤3 cm | 134 | 59 (44.0) | 75 (56.0) | 0.262 | 165 | 82 (49.7) | 83 (50.3) | 0.845 |
| >3 cm | 21 | 12 (57.19 | 9 (42.9) |  | 31 | 16 (51.6) | 15 (48.4) |  |

*P-values were calculated using the Chi-square test. Low: <1% positive cells. High: ≥1% positive cells. CEA=carcinoembryonic antigen, CRP=C‐reactive protein.

# Supplementary Table 6. Correlations between PD-L1^+^ tumour cells in lung metastases and primary tumours, respectively, and clinicopathological factors.

| **PD-L1 ^TC^** | **Primary tumour** | | | | **Lung metastases** | | | |
| --- | --- | --- | --- | --- | --- | --- | --- | --- |
| **Factor** | **N** | **Low** | **High** | **p-value*** | **N** | **Low** | **High** | **p-value*** |
| *Age at first pulmonary metastasectomy* | 167 |  |  |  | 206 |  |  |  |
| ≤60 years | 38 | 37 (97.4) | 1 (2.6) | 0.881 | 48 | 44 (91.7) | 4 (8,3) | 0.125 |
| >60 years | 129 | 153 (96.8) | 4 (3.2) |  | 158 | 153 (96.8) | 5 (3.2) |  |
| *Gender* | 167 |  |  |  | 206 |  |  |  |
| Male | 97 | 95 (97.95) | 2 (2.95) | 0.405 | 122 | 118 (96.7) | 4 (3.3) | 0.356 |
| Female | 70 | 67 (95.7) | 3 (4.3) |  | 84 | 79 (94.0) | 5 (6.0) |  |
| *Synchronous metastasis* | 167 |  |  |  | 206 |  |  |  |
| Yes | 40 | 39 (97.5) | 1 (2.5) | 0.833 | 50 | 48 (96.0) | 2 (4.0) | 0.883 |
| No | 127 | 123 (96.9) | 4 (3.1) |  | 156 | 149 (95.5) | 7 (4.5) |  |
| *Disease-free interval* | 167 |  |  |  | 206 |  |  |  |
| ≤24 months | 103 | 101 (98.1) | 2 (1.9) | 0.311 | 120 | 114 (95.0) | 6 (5.0) | 0.601 |
| >24 months | 64 | 61 (95.3) | 3 (4.7) |  | 87 | 86 (96.5) | 3 (3.5) |  |
| *CEA before metastasectomy* | 105 |  |  |  | 124 |  |  |  |
| ≤5 μg /L | 83 | 81 (97.6) | 2 (2.4) | 0.593 | 94 | 90 (95.7) | 4 (4.3) | 0.592 |
| >5 μg /L | 22 | 21 (95.5) | 1 (4.5) |  | 30 | 28 (93.3) | 2 (6.7) |  |
| *CRP before metastasectomy* | 147 |  |  |  | 180 |  |  |  |
| ≤10 mg/L | 133 | 128 (96.2) | 5 (3.8) | 0.460 | 162 | 153 (94.4) | 9 (5.6) | 0.305 |
| >10 mg/L | 14 | 14 (100.0) | 0 (0.0) |  | 18 | 18 (100.0) | 0 (0.0) |  |
| *Location of primary tumour* | 167 |  |  |  | 206 |  |  |  |
| Right colon | 15 | 15 (100.0) | 0 (0.0) | 0.495 | 16 | 15 (93.75) | 1 (6.25) | 0.714 |
| Left colon | 60 | 59 (98.3) | 1 (1.7) |  | 71 | 69 (97.2) | 2 (2.8) |  |
| Rectum | 92 | 88 (95.7) | 4 (4.3) |  | 119 | 113 (95.0) | 6 (5.0) |  |
| *Primary tumour stage* |  |  |  |  |  |  |  |  |
| T stage | 159 |  |  |  | 187 |  |  |  |
| 1 or 2 | 24 | 24 (100.0) | 0 (0.0) | 0.317 | 32 | 31 (96.9) | 1 (3.1) | 0.564 |
| 3 | 110 | 105 (95.5) | 5 (4.5) |  | 127 | 122 (96.1) | 5 (3.9) |  |

| 4 | 25 | 25 (100.0) | 0 (0.0) |  | 28 | 28 (100.0) | 0 (0.0) |  |
| --- | --- | --- | --- | --- | --- | --- | --- | --- |
| N stage | 162 |  |  |  | 194 |  |  |  |
| 0 | 60 | 58 (96.7) | 2 (3.3) | 0.621 | 81 | 78 (96.3) | 3 (3.7) | 0.658 |
| 1 | 62 | 61 (98.4) | 1 (1.6) |  | 70 | 66 (94.3) | 4 (5.7) |  |
| 2 | 40 | 38 (95.0) | 2 (5.0) |  | 43 | 42 (97.7) | 1 (2.3) |  |
| *CEA before surgery of primary tumour* | 84 |  |  |  | 91 |  |  |  |
| ≤5 ng/mL | 51 | 48 (94.1) | 3 (5.9) | 0.973 | 54 | 53 (98.1) | 1 (1.9) | 0.065 |
| >5 ng/mL | 33 | 31 (93.9) | 2 (6.1) |  | 37 | 33 (89.2) | 4 (10.8) |  |
| *Surgery for liver metastasis* | 167 |  |  |  | 206 |  |  |  |
| Yes | 57 | 55 (96.5) | 2 (3.5) | 0.779 | 141 | 134 (95.0) | 7 (5.0) | 0.538 |
| No | 110 | 107 (97.3) | 3 (2.7) |  | 65 | 63 (96.9) | 2 (3.1) |  |
| *Number of lung metastases* | 167 |  |  |  | 206 |  |  |  |
| 1 | 116 | 112 (96.6) | 4 (3.5) | 0.603 | 143 | 135 (94.4) | 8 (5.6) | 0.195 |
| ≥2 | 51 | 50 (98.0) | 1 (2.0) |  | 63 | 62 (98.4) | 1 (1.6) |  |
| *Size of lung metastases* | 158 |  |  |  | 196 |  |  |  |
| ≤3 cm | 136 | 133 (97.8) | 3 (2.2) | 0.517 | 165 | 157 (95.2) | 8 (4.8) | 0.692 |
| >3 cm | 22 | 21 (95.5 | 1 (4.5) |  | 31 | 30 (96.8) | 1 (3.2) |  |

*P-values were calculated using the Chi-square test. Low: <1% positive cells. High: ≥1% positive cells. CEA=carcinoembryonic antigen, CRP=C‐reactive protein.

# Supplementary Table 7. Correlations between the presence of TLLSs in lung metastases and primary tumours, respectively, and clinicopathological factors.

| **TLLS** | **Primary tumour** | | | | **Lung metastases** | | | |
| --- | --- | --- | --- | --- | --- | --- | --- | --- |
| **Factor** | **N** | **Low** | **High** | **p-value*** | **N** | **Low** | **High** | **p-value*** |
| *Age at first pulmonary metastasectomy* | 168 |  |  |  | 208 |  |  |  |
| ≤60 years | 39 | 38 (97.4) | 1 (2.6) | 0.377 | 48 | 40 (83.3) | 8 (16.7) | 0.696 |
| >60 years | 129 | 121 (93.8) | 8 (6.2) |  | 160 | 137 (85.6) | 23 (14.4) |  |
| *Gender* | 168 |  |  |  | 208 |  |  |  |
| Male | 97 | 92 (94.8) | 5 (5.2) | 0.892 | 123 | 104 (84.6) | 19 (15.4) | 0.791 |
| Female | 71 | 67 (94.4) | 4 (5.6) |  | 85 | 73 (85.9) | 12 (14.1) |  |
| *Synchronous metastasis* | 16 |  |  |  | 208 |  |  |  |
| Yes | 39 | 39 (100.0) | 0 (0.0) | 0.090 | 50 | 86 (32.0) | 7 (14.0) | 0.873 |
| No | 129 | 120 (93.0) | 9 (7.0) |  | 158 | 134 (84.8) | 24 (15.2) |  |
| *Disease-free interval* | 168 |  |  |  | 208 |  |  |  |
| ≤24 months | 102 | 96 (94.1) | 6 (5.9) | 0.707 | 121 | 105 (86.8) | 16 (13.2) | 0.422 |
| >24 months | 66 | 63 (95.5) | 3 (4.5) |  | 87 | 72 (82.8) | 15 (17.2) |  |
| *CEA before metastasectomy* | 105 |  |  |  | 125 |  |  |  |
| ≤5 μg /L | 83 | 76 (91.6) | 7 (8.4) | 0.541 | 95 | 82 (86.3) | 13 (13.7) | 0.117 |
| >5 μg /L | 22 | 21 (95.5) | 1 (4.5) |  | 30 | 29 (96.7) | 1 (3.3) |  |
| *CRP before metastasectomy* | 149 |  |  |  | 182 |  |  |  |
| ≤10 mg/L | 135 | 127 (94.1) | 8 (5.9) | 0.856 | 164 | 138 (84.1) | 26 (15.9) | 0.068 |
| >10 mg/L | 14 | 13 (92.9) | 1 (7.1) |  | 18 | 18 (100.0) | 0 (0.0) |  |
| *Location of primary tumour* | 168 |  |  |  | 208 |  |  |  |
| Right colon | 16 | 16 (100.0) | 0 (0.0) | 0.315 | 16 | 15 (93.8) | 1 (6.3) | 0.539 |
| Left colon | 60 | 58 (96.7) | 2 (3.3) |  | 71 | 61 (85.9) | 10 (14.1) |  |
| Rectum | 92 | 85 (92.4) | 7 (7.6) |  | 121 | 101 (83.5) | 20 (16.5) |  |
| *Primary tumour stage* |  |  |  |  |  |  |  |  |
| T stage | 158 |  |  |  | 186 |  |  |  |
| 1 or 2 | 24 | 23 (95.8) | 1 (4.2) | 0.331 | 32 | 30 (93.8) | 2 (6.3) | 0.206 |
| 3 | 110 | 102 (92.7) | 8 (7.3) |  | 129 | 106 (82.2) | 23 (17.8) |  |
| 4 | 26 | 26 (100.0) | 0 (0.0) |  | 28 | 25 (89.3) | 3 (10.7) |  |

| N stage | 163 |  |  |  | 196 |  |  |  |
| --- | --- | --- | --- | --- | --- | --- | --- | --- |
| 0 | 60 | 57 (95.0) | 3 (5.0) | 0.060 | 81 | 72 (88.9) | 9 (11.1) | 0.329 |
| 1 | 63 | 62 (98.4) | 1 (1.6) |  | 72 | 62 (86.1) | 10 (13.9) |  |
| 2 | 40 | 35 (87.5) | 5 (12.5) |  | 43 | 34 (79.1) | 9 (20.9) |  |
| *CEA before surgery of primary tumour* | 83 |  |  |  | 93 |  |  |  |
| ≤5 ng/mL | 51 | 46 (90.2) | 5 (9.8) | 0.571 | 55 | 46 (83.6) | 9 (16.4) | 0.036 |
| >5 ng/mL | 32 | 30 (93.75) | 2 (6.25) |  | 38 | 37 (97.4) | 1 (2.6) |  |
| *Surgery for liver metastasis* | 168 |  |  |  | 208 |  |  |  |
| Yes | 58 | 55 (94.8) | 3 (5.2) | 0.938 | 66 | 61 (92.4) | 5 (7.6) | 0.043 |
| No | 110 | 104 (94.5) | 6 (5.5) |  | 142 | 116 (81.7) | 26 (18.3) |  |
| *Number of lung metastases* | 168 |  |  |  | 208 |  |  |  |
| 1 | 117 | 111 (94.9) | 6 (5.1) | 0.842 | 144 | 120 (83.3) | 24 (16.7) | 0.248 |
| ≥2 | 51 | 48 (94.1) | 3 (5.9%) |  | 64 | 57 (89.1) | 7 (10.9) |  |
| *Size of lung metastases* | 158 |  |  |  | 198 |  |  |  |
| ≤3 cm | 136 | 128 (44.0) | 8 (56.0) | 0.243 | **167** | **139 (83.2)** | **28 (16.8)** | **0.050** |
| >3 cm | 22 | 22 (100.0) | 0 (0.0) |  | **31** | **30 (96.8)** | **1 (3.2)** |  |

*P-values were calculated using the Chi-square test. TLLS=tertiary lymphoid-like structures. Low: no TLLS, high: ≥1 TLLS. CEA=carcinoembryonic antigen, CRP=C‐ reactive protein.

# Supplementary Table 8. Cox proportional hazards regression analysis of OS following PM, based on immune marker expression in lung metastases.

|  | Univariable | | Multivariable | |
| --- | --- | --- | --- | --- |
| Factor | N | HR (95% CI) | N | HR (95% CI) |
| CD3 high vs low | 156, 52 | **0.69 (0.47-1.00)** | 129, 43 | 0.79 (0.50-1.23) |
| CD8 high vs low | 87, 120 | 1.23 (0.87-1.74) | 69, 102 | 1.47 (0.99-2.20) |
| FoxP3 high vs low | 155, 51 | 0.87 (0.59-1.30) | 132, 38 | 1.18 (0.71-1.96) |
| CD20 high vs low | 72, 136 | 0.71(0.49-1.04) | 60, 112 | 0.74 (0.48-1.15) |
| PD-LI ^IC^ high vs low | 102, 104 | 0.88 (0.62-1.24) | 86, 84 | 0.80 (0.54-1.20) |
| TLLS any vs none | 31, 177 | 0.62 (0.36-1.06) | 22, 150 | 0.79 (0.42-1.50) |

Adjusted for age (continuous), >1 metastasis, size of metastasis (continuous), DFI <24 months vs ≥24 months, N2 vs N0-N1, CEA > 5ug/l before PM, neoadjuvant vs no neoadjuvant therapy before PM, adjuvant vs no adjuvant chemotherapy after PM.

# Supplementary Table 9. Cox proportional hazards regression analysis of OS following PM, based on immune marker expression in primary tumours.

|  | Univariable | | Multivariable | |
| --- | --- | --- | --- | --- |
| Factor | N | HR (95% CI) | N | HR (95% CI) |
| CD3 high vs low | 69, 99 | 0.856 (0.58-1.26) | 129, 43 | 0.79 (0.50-1.23) |
| CD8 high vs low | 24, 144 | 0.99 (0.59-1.66) | 69, 102 | 1.47 (0.99-2.20) |
| FoxP3 high vs low | 100, 68 | 1.07 (0.73-1.58) | 132, 38 | 1.18 (0.71-1.96) |
| CD20 high vs low | 17,151 | 1.27 (0.70-2.3) | 60, 112 | 0.74 (0.48-1.15) |
| PD-L1^IC^ high vs low | 51, 116 | 0.82 (0.54-1.26) | 86, 84 | 0.80 (0.54-1.20) |
| TLLS any vs none | 9, 159 | 1.39 (0.61-3.17) | 22, 150 | 0.79 (0.42-1.50) |

Adjusted for age (continuous), >1 metastasis, size of metastasis (continuous), DFI <24 months vs ≥24 months, N2 vs N0-N1, CEA > 5ug/l before PM, neoadjuvant vs no neoadjuvant therapy before PM, adjuvant vs no adjuvant chemotherapy after PM
