# Supplementary material for: Tumor‐Infiltrating Immune Cells Are More Abundant in Lung Metastases From Colorectal Cancer Than in Paired Primary Tumors and Their Prognostic Value Depends on Adjuvant Chemotherapy
Source: Cancer Med. 2026 Mar 20;15(3):e71739. doi: 10.1002/cam4.71739 (PMC13093549; doi:10.1002/cam4.71739)
Supplement: Supplementary file 2 — Data S2: Supplementary Figures. [file CAM4-15-e71739-s001.docx]

# SUPPLEMENTARY FIGURES

**Supplementary Figure 1. FoxP3/CD3 double staining and Distribution of PD-L1 tumour cell expression across different tumour sites**. A) Double IHC staining of FoxP3 (nuclear) and CD3 (cytoplasmic/membrane) showing a representative tumor with a typical CD3^+^FoxP3^+^ and CD3^+^FoxP3^-^ staining pattern of T-cells (CD3^+^FoxP3^-^, Blue arrows; CD3^+^FoxP3^+^; Green arrows; CD3^low^FoxP3^+^, Yellow arrows; CD3^-^FoxP3^+^, Black arrows) where CD3^-^FoxP3^+^ cells were only occasional and in minority (< 5 cells). B) PD-L1 tumour cell expression in primary tumours (prim1), initial/largest lung metastases (lung1), and initial/largest liver metastases (liver1). Lung metastases are stratified by neoadjuvant chemotherapy status (no neo vs. neo). Comparisons between primary tumors and metastases were performed using the Wilcoxon signed-rank test; differences between lung subgroups were assessed using the Mann-Whitney U test. P-values indicate statistical significance (ns=not significant).

**Supplementary figure 2. Kaplan-Meier curves illustrating OS in the entire cohort, stratified by all annotated categories of immune cell infiltration and the number of TLLSs in lung metastases**. Survival is shown for A) CD3, B) CD8, C) CD20, D) FoxP3, E) PD-L1 expression on immune cells, F) PD-L1 expression on tumour cells, and G) TLLS count. In the number at risk tables for CD3 and PD-L1^TC^, the strata with no cases were omitted.

**Supplementary figure 3. Kaplan-Meier curves illustrating OS in the entire cohort, stratified by all annotated categories of immune cell infiltration in lung metastases, further stratified by adjuvant chemotherapy status.** Survival is shown for CD3 expression in A) untreated and B) treated patients; for CD8 in C) untreated and D) treated patients; for FoxP3 in E) untreated and F) treated patients; for CD20 in G) untreated and H) treated patients. In the number at risk tables for CD3, the strata with no cases were omitted.

**Supplementary figure 4. Kaplan-Meier curves illustrating OS in the entire cohort, stratified by all annotated categories of immune cell infiltration in lung metastases and the number of TLLSs, further stratified by adjuvant chemotherapy status.** Survival is shown for PD-L1 immune cell expression in A) untreated and B) treated patients; for PD-L1 tumour cell expression in C) untreated and D) treated patients; and for TLLS count in E) untreated and F) treated patients. In the number at risk tables for PD-L1^TC^, the strata with no cases were omitted.

**Supplementary figure 5. Kaplan-Meier curves illustrating OS in the entire cohort, stratified by all annotated categories of immune cell infiltration and the number of TLLSs in primary tumours**. Survival is shown for A) CD3, B) CD8, C) CD20, D) FoxP3,

E) PD-L1 expression on immune cells, F) PD-L1 expression on tumour cells, and G) TLLS count. In the number at risk tables for PD-L1^TC^, the strata with no cases were omitted.

## Supplementary figure 6. Kaplan-Meier curves illustrating OS in the entire cohort, stratified by all annotated categories of immune cell infiltration in primary tumours,

**further stratified by adjuvant chemotherapy status.** Survival is shown for CD3 expression in A) untreated (A) and treated (B) group; CD8 in untreated (C) and treated (D) group; FoxP3 in untreated (E) and treated (F) group; CD20 in untreated (G) and treated (H) group.

**Supplementary figure 7. Kaplan-Meier curves illustrating OS in the entire cohort, stratified by all annotated categories of immune cell infiltration in primary tumours and the number of TLLSs, further stratified by adjuvant chemotherapy status**. Survival is shown for PD-L1 immune cell expression in A) untreated and B) treated patients; for PD-L1 tumour cell expression in C) untreated and D) treated patients; and for TLLS count in E) untreated and F) treated patients. In the number at risk tables for PD-L1^TC^ and TLLS, the strata with no cases were omitted.

**Supplementary figure 8. Kaplan-Meier curves illustrating OS in the entire cohort, stratified by all annotated categories of immune cell infiltration and the number of TLLSs in liver metastases.** Survival is shown for A) CD3, B) CD8, C) CD20, D) FoxP3, E) PD-L1^IC^, F) PD-L1^TC^, and G) TLLS count. In the number at risk tables for PD-L1^TC^ and TLLS, the strata with no cases were omitted.

# (A)

## FoxP3 (nuclear)

Supplementary Figure 1

**CD3 (cytoplasmic and membrane)**


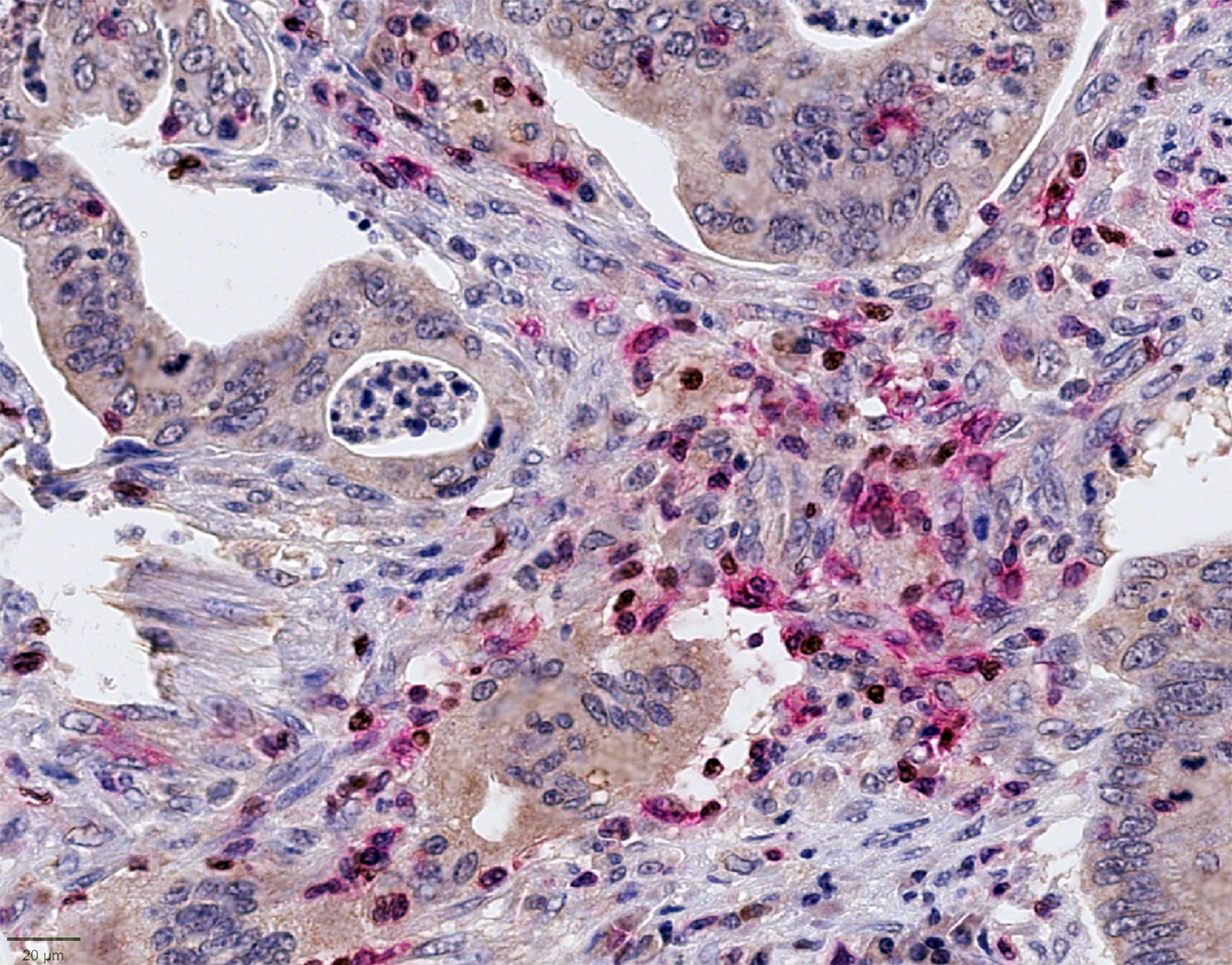

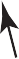

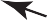

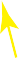

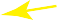

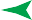

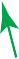

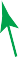

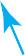

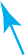


#
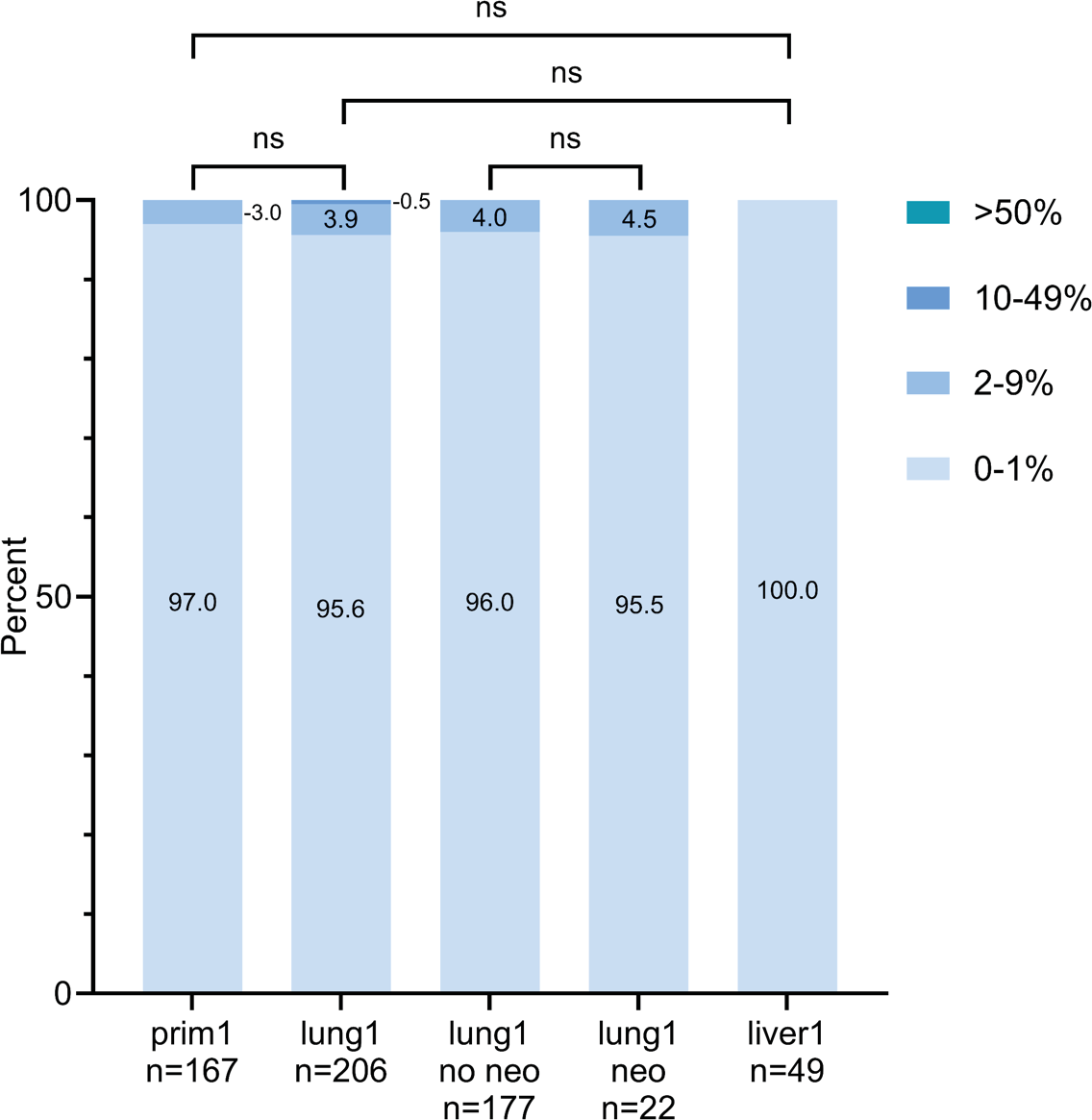
(B)


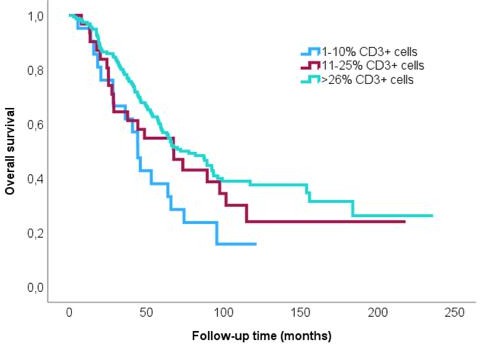


| Number at risk |  |  |  |  |  |  |
| --- | --- | --- | --- | --- | --- | --- |
| 1-10% CD3+ cell s | 21 | 9 | 2 | 0 | 0 | 0 |
| 11-25% CD3+ cell s | 31 | 17 | 8 | 3 | 2 | 0 |
| >26% CD3+ cel ls | 156 | 103 | 39 | 14 | 4 | 0 |

| Number at risk |  |  |  |  |  |  |
| --- | --- | --- | --- | --- | --- | --- |
| 0% CD8+ cell s | 33 | 17 | 6 | 0 | 0 | 0 |
| 1-10% CD8+ cell s | 87 | 58 | 24 | 7 | 3 | 0 |
| 11-25% CD8+ cell s | 57 | 33 | 8 | 5 | 1 | 0 |
| >26% CD8+ cel ls | 30 | 21 | 11 | 5 | 2 | 0 |

**
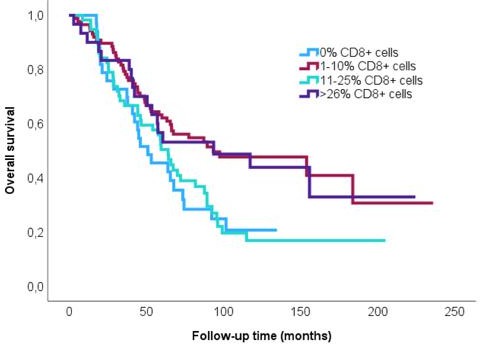
(C) (D)**

Supplementary Figure 2

**
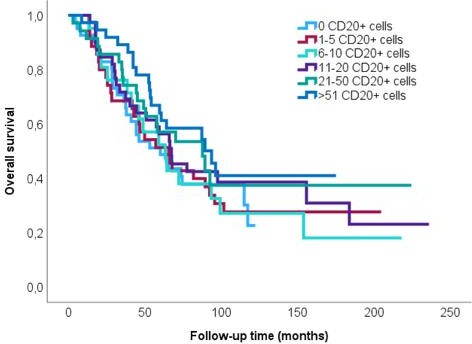

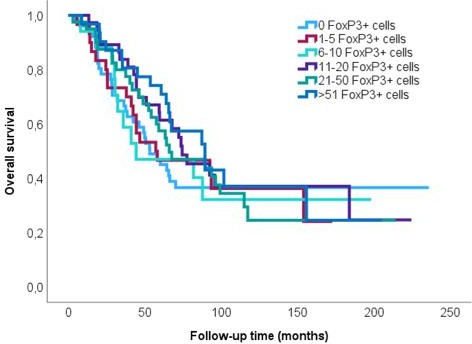
**

| Number at risk |  |  |  |  |  |  |
| --- | --- | --- | --- | --- | --- | --- |
| 0 CD20+ cells | 41 | 22 | 10 | 0 | 0 | 0 |
| 1-5 CD20+ cells | 35 | 19 | 10 | 3 | 1 | 0 |
| 6-10 CD20+ cells | 21 | 12 | 5 | 3 | 1 | 0 |
| 11-20 CD20+ cells | 39 | 25 | 10 | 5 | 3 | 0 |
| 21-50 CD20+ cells | 35 | 23 | 6 | 3 | 1 | 0 |
| >51 CD20+ cells | 37 | 28 | 8 | 3 | 0 | 0 |

| Number at risk |  |  |  |  |  |  |
| --- | --- | --- | --- | --- | --- | --- |
| 0 Fox P3+ cel ls | 51 | 28 | 11 | 4 | 2 | 0 |
| 1-5 Fox P3+ cel ls | 30 | 16 | 7 | 3 | 0 | 0 |
| 6-10 Fox P3+ cel ls | 17 | 8 | 3 | 1 | 0 | 0 |
| 11-20 Fox P3+ cel ls | 37 | 25 | 9 | 4 | 2 | 0 |
| 21-50 Fox P3+ cel ls | 40 | 27 | 11 | 2 | 1 | 0 |
| >51 Fox P3+ cells | 31 | 24 | 8 | 3 | 1 | 0 |

**
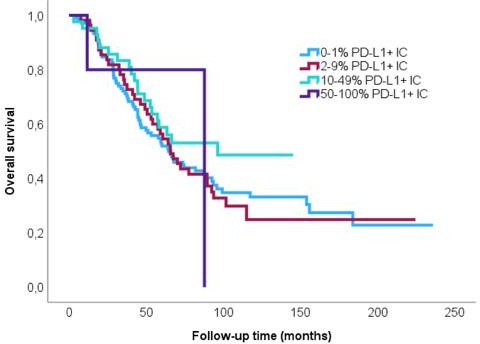

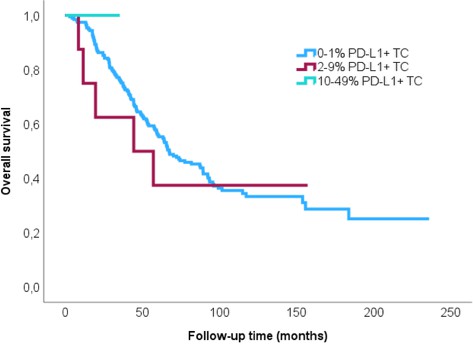
(E) (F)**

| Number at risk |  |  |  |  |  |  |
| --- | --- | --- | --- | --- | --- | --- |
| 0-1% P D-L1+ IC | 104 | 60 | 25 | 13 | 4 | 0 |
| 2-9% P D-L1+ IC | 55 | 36 | 14 | 4 | 2 | 0 |
| 10-49% P D-L1+ IC | 42 | 28 | 10 | 0 | 0 | 0 |
| 50-100% P D-L1+ IC | 5 | 4 | 0 | 0 | 0 | 0 |

| Number at risk |  |  |  |  |  |  |
| --- | --- | --- | --- | --- | --- | --- |
| 0-1% P D-L1+ TC | 197 | 124 | 46 | 16 | 6 | 0 |
| 2-9% P D-L1+ TC | 8 | 4 | 3 | 1 | 0 | 0 |
| 10-49% P D-L1+ TC | 1 | 0 | 0 | 0 | 0 | 0 |

**
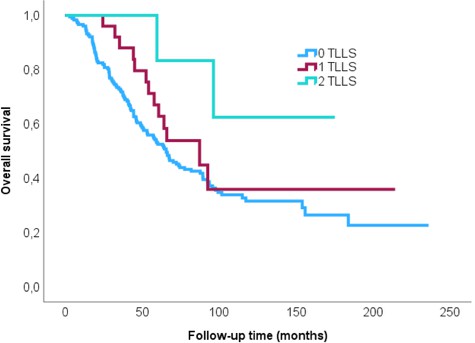
(G)**

| Number at risk |  |  |  |  |  |  |
| --- | --- | --- | --- | --- | --- | --- |
| 0 TLLS | 177 | 104 | 42 | 14 | 5 | 0 |
| 1 TLLS | 25 | 19 | 4 | 2 | 1 | 0 |
| 2 TLLS | 6 | 6 | 3 | 1 | 0 | 0 |


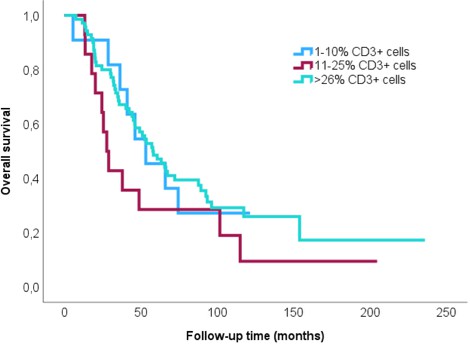

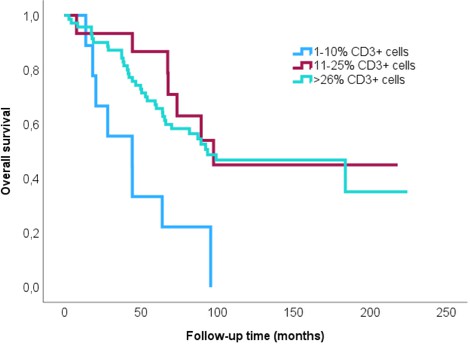


| Number at risk |  |  |  |  |  |  |
| --- | --- | --- | --- | --- | --- | --- |
| 1-10% CD3+ cell s | 11 | 6 | 2 | 0 | 0 | 0 |
| 11-25% CD3+ cell s | 14 | 4 | 3 | 1 | 1 | 0 |
| >26% CD3+ cel ls | 70 | 40 | 12 | 3 | 2 | 0 |

| Number at risk |  |  |  |  |  |  |
| --- | --- | --- | --- | --- | --- | --- |
| 1-10% CD3+ cell s | 9 | 3 | 0 | 0 | 0 | 0 |
| 11-25% CD3+ cell s | 15 | 13 | 5 | 2 | 1 | 0 |
| >26% CD3+ cel ls | 70 | 51 | 24 | 10 | 2 | 0 |

**(C) (D)**

Supplementary Figure 3

**
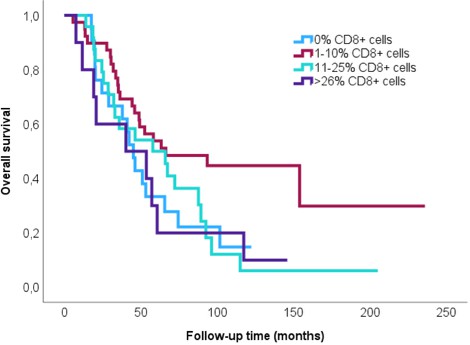

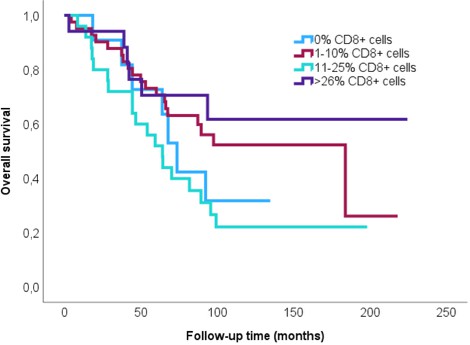
**

| Number at risk |  |  |  |  |  |  |
| --- | --- | --- | --- | --- | --- | --- |
| 0% CD8+ cell s | 21 | 9 | 3 | 0 | 0 | 0 |
| 1-10% CD8+ cell s | 39 | 23 | 10 | 3 | 2 | 0 |
| 11-25% CD8+ cell s | 24 | 13 | 2 | 1 | 0 | 0 |
| >26% CD8+ cel ls | 10 | 5 | 2 | 0 | 0 | 0 |

| Number at risk |  |  |  |  |  |  |
| --- | --- | --- | --- | --- | --- | --- |
| 0% CD8+ cell s | 11 | 8 | 3 | 0 | 0 | 0 |
| 1-10% CD8+ cell s | 41 | 31 | 14 | 4 | 1 | 0 |
| 11-25% CD8+ cell s | 25 | 15 | 5 | 4 | 0 | 0 |
| >26% CD8+ cel ls | 17 | 13 | 7 | 4 | 2 | 0 |

**
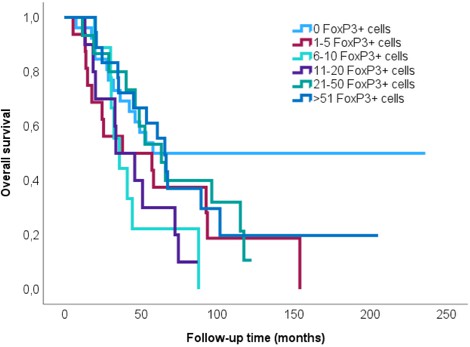

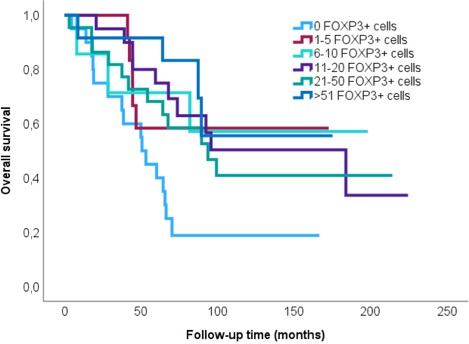
(E) (F)**

| Number at risk |  |  |  |  |  |  |
| --- | --- | --- | --- | --- | --- | --- |
| 0 Fox P3+ cel ls | 26 | 15 | 8 | 2 | 2 | 0 |
| 1-5 Fox P3+ cel ls | 16 | 8 | 2 | 1 | 0 | 0 |
| 6-10 Fox P3+ cel ls | 9 | 2 | 0 | 0 | 0 | 0 |
| 11-20 Fox P3+ cel ls | 10 | 4 | 0 | 0 | 0 | 0 |
| 21-50 Fox P3+ cel ls | 15 | 9 | 4 | 0 | 0 | 0 |
| >51 Fox P3+ cells | 18 | 12 | 3 | 1 | 0 | 0 |

| Number at risk |  |  |  |  |  |  |
| --- | --- | --- | --- | --- | --- | --- |
| 0 Fox P3+ cel ls | 20 | 11 | 2 | 2 | 0 | 0 |
| 1-5 Fox P3+ cel ls | 12 | 7 | 5 | 2 | 0 | 0 |
| 6-10 Fox P3+ cel ls | 7 | 5 | 3 | 1 | 0 | 0 |
| 11-20 Fox P3+ cel ls | 20 | 16 | 8 | 4 | 2 | 0 |
| 21-50 Fox P3+ cel ls | 22 | 16 | 7 | 2 | 1 | 0 |
| >51 Fox P3+ cells | 12 | 11 | 4 | 1 | 0 | 0 |

**(G) (H)**


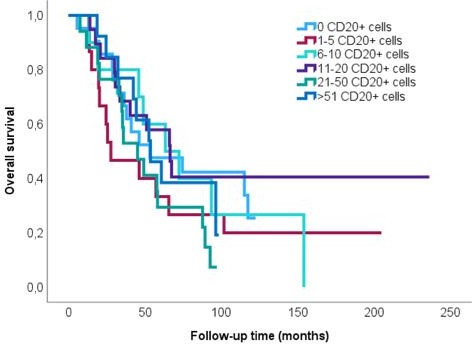

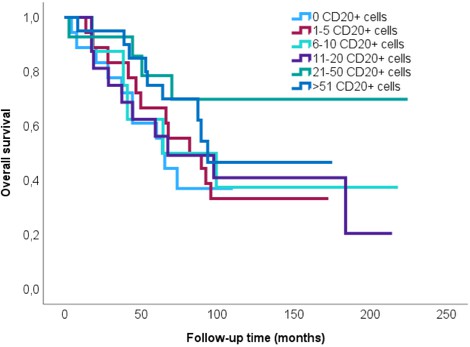


| Number at risk |  |  |  |  |  |  |
| --- | --- | --- | --- | --- | --- | --- |
| 0 CD20+ cells | 21 | 11 | 7 | 0 | 0 | 0 |
| 1-5 CD20+ cells | 15 | 6 | 4 | 1 | 0 | 0 |
| 6-10 CD20+ cells | 10 | 6 | 2 | 1 | 0 | 0 |
| 11-20 CD20+ cells | 19 | 12 | 4 | 2 | 2 | 0 |
| 21-50 CD20+ cells | 17 | 7 | 0 | 0 | 0 | 0 |
| >51 CD20+ cells | 13 | 8 | 0 | 0 | 0 | 0 |

| Number at risk |  |  |  |  |  |  |
| --- | --- | --- | --- | --- | --- | --- |
| 0 CD20+ cells | 18 | 11 | 3 | 0 | 0 | 0 |
| 1-5 CD20+ cells | 18 | 12 | 6 | 2 | 0 | 0 |
| 6-10 CD20+ cells | 8 | 5 | 3 | 2 | 1 | 0 |
| 11-20 CD20+ cells | 16 | 10 | 5 | 2 | 1 | 0 |
| 21-50 CD20+ cells | 14 | 12 | 6 | 3 | 1 | 0 |
| >51 CD20+ cells | 20 | 17 | 6 | 3 | 0 | 0 |

Supplementary Figure 4

**
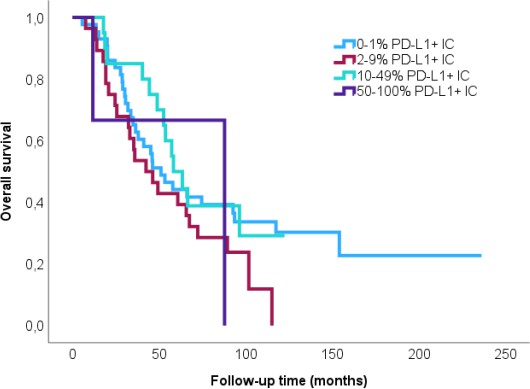

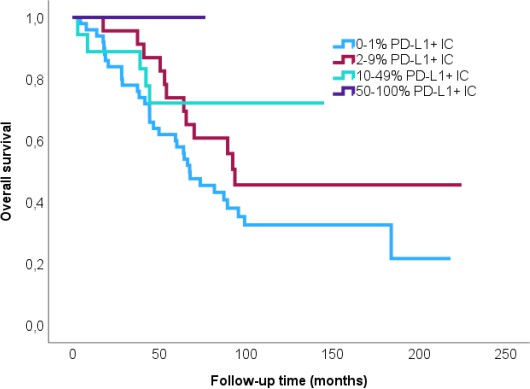
(A) (B)**

| Number at risk |  |  |  |  |  |  |
| --- | --- | --- | --- | --- | --- | --- |
| 0-1% PD-L1+ IC | 43 | 22 | 11 | 4 | 3 | 0 |
| 2-9% PD-L1+ IC | 28 | 12 | 4 | 0 | 0 | 0 |
| 10-49% PD-L1+ IC | 20 | 14 | 2 | 0 | 0 | 0 |
| 50-100% PD-L1+ IC | 3 | 2 | 0 | 0 | 0 | 0 |

| Number at risk |  |  |  |  |  |  |
| --- | --- | --- | --- | --- | --- | --- |
| 0-1% PD-L1+ IC | 50 | 31 | 12 | 8 | 1 | 0 |
| 2-9% PD-L1+ IC | 23 | 20 | 9 | 4 | 2 | 0 |
| 10-49% PD-L1+ IC | 18 | 13 | 8 | 0 | 0 | 0 |
| 50-100% PD-L1+ IC | 1 | 1 | 0 | 0 | 0 | 0 |

**(C) (D)**

**
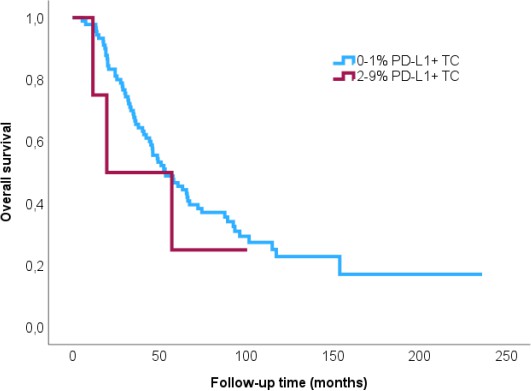

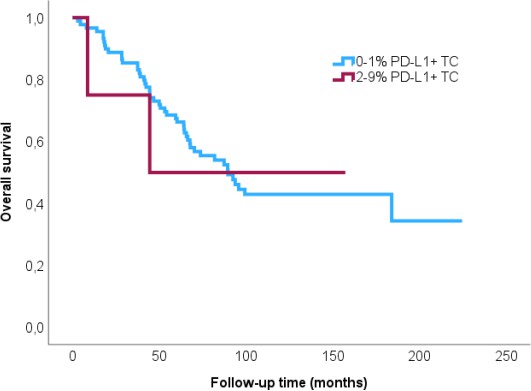
**

| Number at risk |  |  |  |  |  |  |
| --- | --- | --- | --- | --- | --- | --- |
| 0-1% PD-L1+ TC | 90 | 48 | 16 | 4 | 3 | 0 |
| 2-9% PD-L1+ TC | 4 | 2 | 1 | 0 | 0 | 0 |

| Number at risk |  |  |  |  |  |  |
| --- | --- | --- | --- | --- | --- | --- |
| 0-1% PD-L1+ TC | 89 | 64 | 27 | 11 | 3 | 0 |
| 2-9% PD-L1+ TC | 4 | 2 | 2 | 1 | 0 | 0 |

**(E) (F)**

**
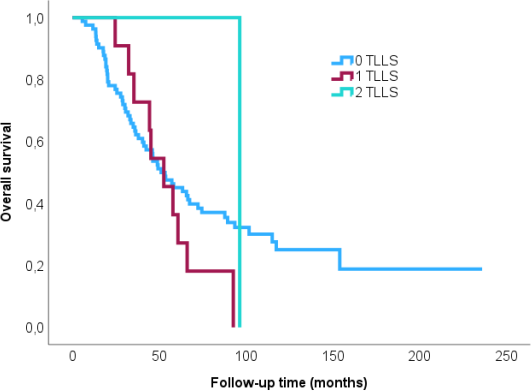

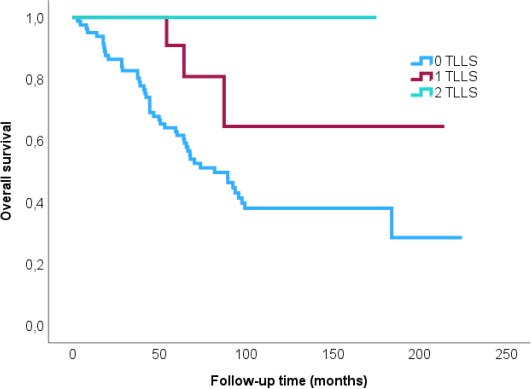
**

| Number at risk |  |  |  |  |  |  |
| --- | --- | --- | --- | --- | --- | --- |
| 0 TLLS | 82 | 42 | 17 | 4 | 3 | 0 |
| 1 TLLS | 11 | 6 | 0 | 0 | 0 | 0 |
| 2 TLLS | 2 | 2 | 1 | 0 | 0 | 0 |

| Number at risk |  |  |  |  |  |  |
| --- | --- | --- | --- | --- | --- | --- |
| 0 TLLS | 81 | 54 | 23 | 9 | 2 | 0 |
| 1 TLLS | 11 | 11 | 4 | 2 | 1 | 0 |
| 2 TLLS | 2 | 2 | 2 | 2 | 0 | 0 |

| Number at risk |  |  |  |  |  |  |
| --- | --- | --- | --- | --- | --- | --- |
| 0% CD3+ cell s | 8 | 3 | 2 | 0 | 0 | 0 |
| 1-10% CD3+ cell s | 45 | 29 | 13 | 5 | 2 | 0 |
| 11-25% CD3+ cell s | 46 | 23 | 7 | 3 | 3 | 0 |
| >26% CD3+ cel ls | 69 | 48 | 18 | 7 | 1 | 0 |

| Number at risk |  |  |  |  |  |  |
| --- | --- | --- | --- | --- | --- | --- |
| 0% CD8+ cell s | 77 | 41 | 13 | 1 | 0 | 0 |
| 1-10% CD8+ cell s | 67 | 45 | 19 | 10 | 5 | 0 |
| 11-25% CD8+ cell s | 15 | 11 | 4 | 2 | 0 | 0 |
| >26% CD8+ cel ls | 9 | 6 | 4 | 2 | 1 | 0 |

**
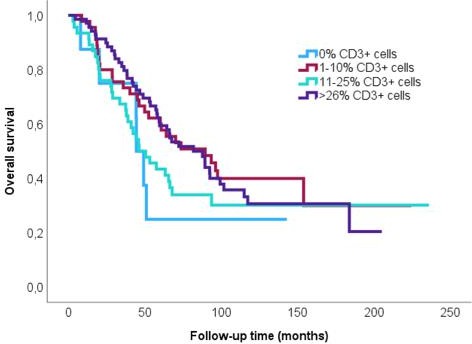

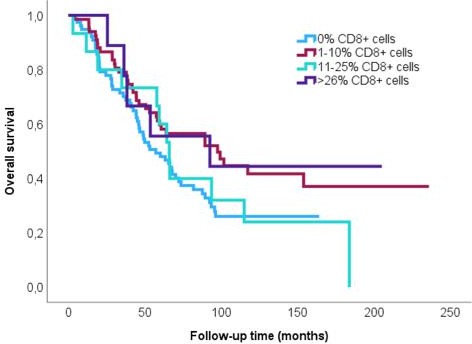
(C) (D)**

Supplementary Figure 5

**
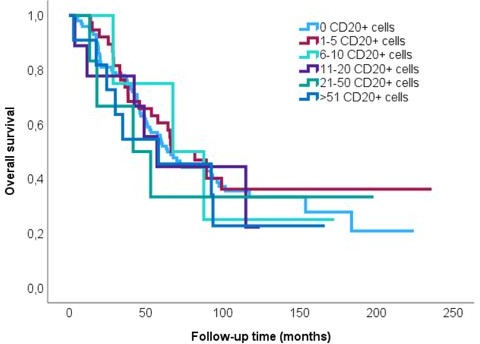

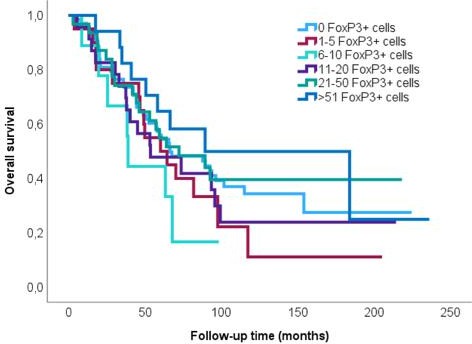
**

| Number at risk |  |  |  |  |  |  |
| --- | --- | --- | --- | --- | --- | --- |
| 0 CD20+ cells | 100 | 61 | 25 | 7 | 3 | 0 |
| 1-5 CD20+ cells | 38 | 25 | 9 | 5 | 3 | 0 |
| 6-10 CD20+ cells | 4 | 3 | 1 | 1 | 0 | 0 |
| 11-20 CD20+ cells | 9 | 5 | 2 | 0 | 0 | 0 |
| 21-50 CD20+ cells | 6 | 3 | 1 | 1 | 0 | 0 |
| >51 CD20+ cells | 11 | 6 | 2 | 1 | 0 | 0 |

| Number at risk |  |  |  |  |  |  |
| --- | --- | --- | --- | --- | --- | --- |
| 0 Fox P3+ cel ls | 68 | 43 | 20 | 6 | 2 | 0 |
| 1-5 Fox P3+ cel ls | 20 | 11 | 2 | 1 | 1 | 0 |
| 6-10 Fox P3+ cel ls | 9 | 4 | 0 | 0 | 0 | 0 |
| 11-20 Fox P3+ cel ls | 23 | 13 | 4 | 2 | 1 | 0 |
| 21-50 Fox P3+ cel ls | 31 | 20 | 8 | 4 | 1 | 0 |
| >51 Fox P3+ cells | 17 | 13 | 6 | 2 | 1 | 0 |

**
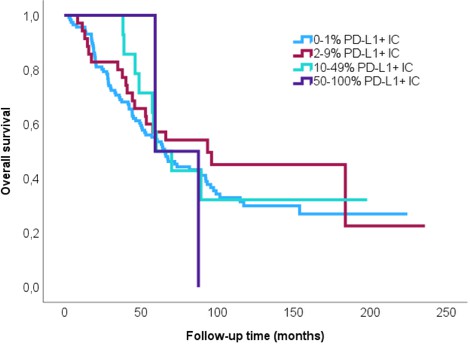

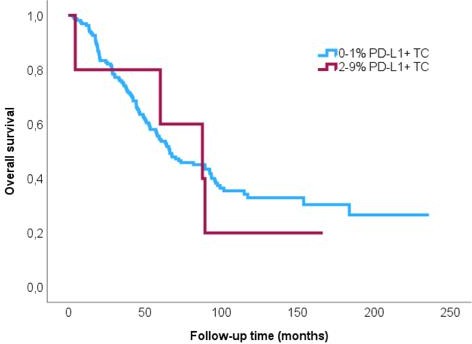
(E) (F)**

| Number at risk |  |  |  |  |  |  |
| --- | --- | --- | --- | --- | --- | --- |
| 0-1% P D-L1+ IC | 116 | 69 | 28 | 11 | 5 | 0 |
| 2-9% P D-L1+ IC | 35 | 23 | 9 | 3 | 1 | 0 |
| 10-49% P D-L1+ IC | 14 | 10 | 3 | 1 | 0 | 0 |
| 50-100% P D-L1+ IC | 2 | 2 | 1 | 0 | 0 | 0 |

| Number at risk |  |  |  |  |  |  |
| --- | --- | --- | --- | --- | --- | --- |
| 0-1% P D-L1+ TC | 162 | 100 | 39 | 14 | 6 | 0 |
| 2-9% P D-L1+ TC | 5 | 4 | 1 | 0 | 0 | 0 |

**(G)**

**
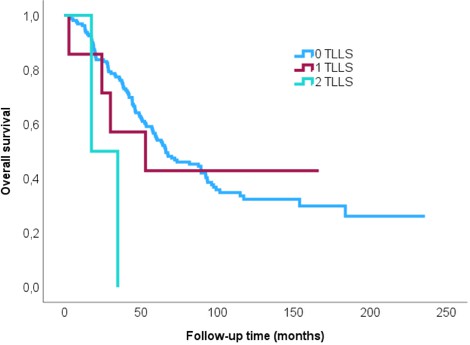
**

| Number at risk |  |  |  |  |  |  |
| --- | --- | --- | --- | --- | --- | --- |
| 0 TLLS | 159 | 99 | 38 | 14 | 6 | 0 |
| 1 TLLS | 7 | 4 | 2 | 1 | 0 | 0 |
| 2 TLLS | 2 | 0 | 0 | 0 | 0 | 0 |

| Number at risk |  |  |  |  |  |  |
| --- | --- | --- | --- | --- | --- | --- |
| 0% CD3+ cell s | 2 | 0 | 0 | 0 | 0 | 0 |
| 1-10% CD3+ cell s | 22 | 17 | 5 | 2 | 0 | 0 |
| 11-25% CD3+ cell s | 28 | 13 | 3 | 2 | 2 | 0 |
| >26% CD3+ cel ls | 37 | 26 | 13 | 6 | 1 | 0 |

| Number at risk |  |  |  |  |  |  |
| --- | --- | --- | --- | --- | --- | --- |
| 0% CD3+ cell s | 5 | 3 | 2 | 0 | 0 | 0 |
| 1-10% CD3+ cell s | 21 | 11 | 7 | 2 | 2 | 0 |
| 11-25% CD3+ cell s | 14 | 7 | 3 | 0 | 0 | 0 |
| >26% CD3+ cel ls | 25 | 19 | 5 | 1 | 0 | 0 |

**
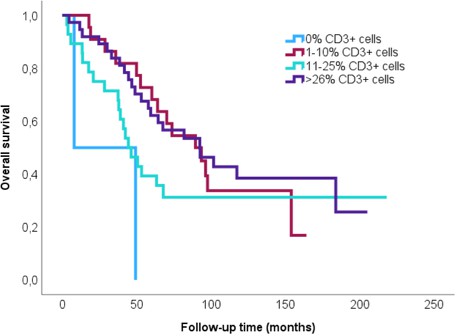

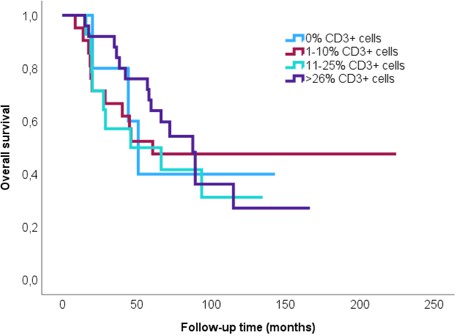
(C) (D)**

Supplementary Figure 6

**
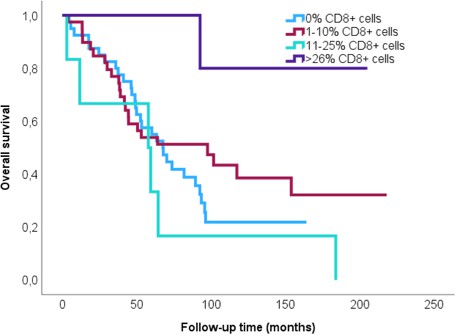

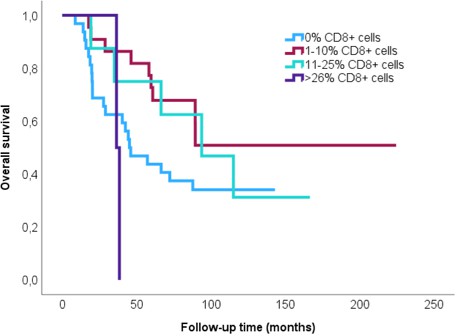
**

| Number at risk |  |  |  |  |  |  |
| --- | --- | --- | --- | --- | --- | --- |
| 0% CD8+ cell s | 40 | 25 | 4 | 0 | 0 | 0 |
| 1-10% CD8+ cell s | 39 | 23 | 12 | 6 | 2 | 0 |
| 11-25% CD8+ cell s | 6 | 3 | 1 | 1 | 0 | 0 |
| >26% CD8+ cel ls | 5 | 5 | 2 | 0 | 0 | 0 |

| Number at risk |  |  |  |  |  |  |
| --- | --- | --- | --- | --- | --- | --- |
| 0% CD8+ cell s | 32 | 15 | 9 | 0 | 0 | 0 |
| 1-10% CD8+ cell s | 22 | 18 | 5 | 2 | 2 | 0 |
| 11-25% CD8+ cell s | 8 | 6 | 3 | 1 | 0 | 0 |
| >26% CD8+ cel ls | 2 | 0 | 0 | 0 | 0 | 0 |

**
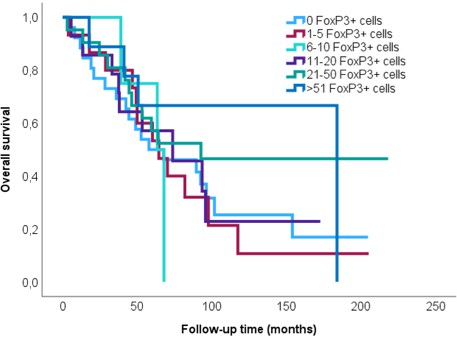

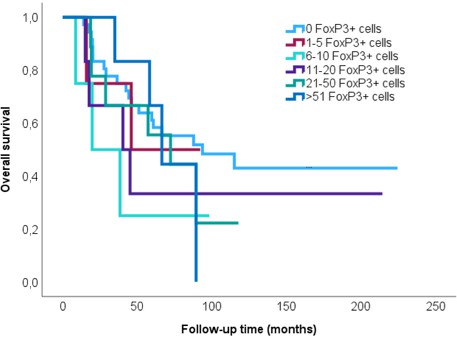
(E) (F)**

| Number at risk |  |  |  |  |  |  |
| --- | --- | --- | --- | --- | --- | --- |
| 0 Fox P3+ cel ls | 26 | 15 | 5 | 3 | 1 | 0 |
| 1-5 Fox P3+ cel ls | 15 | 9 | 2 | 1 | 1 | 0 |
| 6-10 Fox P3+ cel ls | 4 | 3 | 0 | 0 | 0 | 0 |
| 11-20 Fox P3+ cel ls | 14 | 9 | 2 | 1 | 0 | 0 |
| 21-50 Fox P3+ cel ls | 21 | 14 | 7 | 4 | 1 | 0 |
| >51 Fox P3+ cells | 9 | 7 | 5 | 1 | 0 | 0 |

| Number at risk |  |  |  |  |  |  |
| --- | --- | --- | --- | --- | --- | --- |
| 0 Fox P3+ cel ls | 36 | 24 | 14 | 2 | 1 | 0 |
| 1-5 Fox P3+ cel ls | 4 | 2 | 0 | 0 | 0 | 0 |
| 6-10 Fox P3+ cel ls | 4 | 1 | 0 | 0 | 0 | 0 |
| 11-20 Fox P3+ cel ls | 6 | 2 | 2 | 1 | 1 | 0 |
| 21-50 Fox P3+ cel ls | 9 | 6 | 1 | 0 | 0 | 0 |
| >51 Fox P3+ cells | 6 | 5 | 0 | 0 | 0 | 0 |

**(G)**

**(H)**


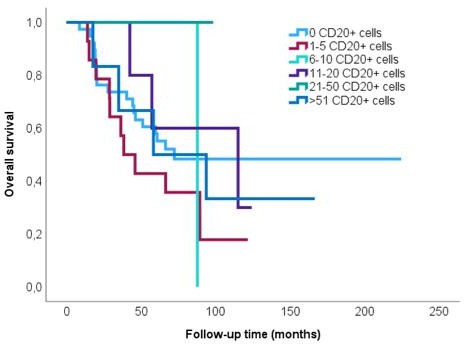

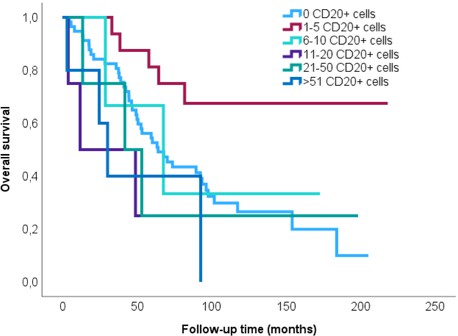


| Number at risk |  |  |  |  |  |  |
| --- | --- | --- | --- | --- | --- | --- |
| 0 CD20+ cells | 57 | 35 | 13 | 4 | 1 | 0 |
| 1-5 CD20+ cells | 16 | 14 | 6 | 4 | 2 | 0 |
| 6-10 CD20+ cells | 3 | 2 | 1 | 1 | 0 | 0 |
| 11-20 CD20+ cells | 4 | 1 | 0 | 0 | 0 | 0 |
| 21-50 CD20+ cells | 4 | 2 | 1 | 1 | 0 | 0 |
| >51 CD20+ cells | 5 | 2 | 0 | 0 | 0 | 0 |

| Number at risk |  |  |  |  |  |  |
| --- | --- | --- | --- | --- | --- | --- |
| 0 CD20+ cells | 38 | 24 | 11 | 2 | 2 | 0 |
| 1-5 CD20+ cells | 14 | 6 | 2 | 0 | 0 | 0 |
| 6-10 CD20+ cells | 1 | 1 | 0 | 0 | 0 | 0 |
| 11-20 CD20+ cells | 5 | 4 | 2 | 0 | 0 | 0 |
| 21-50 CD20+ cells | 1 | 1 | 0 | 0 | 0 | 0 |
| >51 CD20+ cells | 6 | 4 | 2 | 0 | 0 | 0 |

Supplementary Figure 7

**
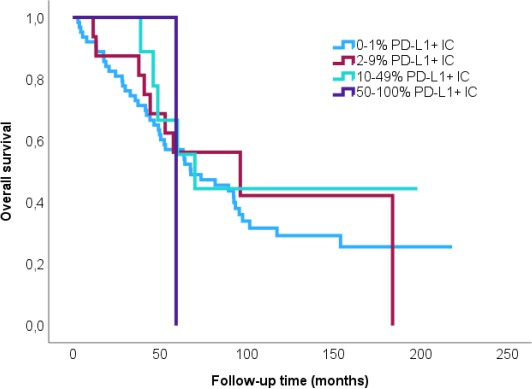

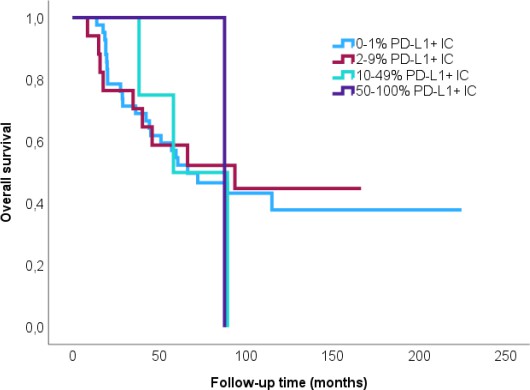
(A) (B)**

| Number at risk |  |  |  |  |  |  |
| --- | --- | --- | --- | --- | --- | --- |
| 0-1% PD-L1+ IC | 63 | 39 | 15 | 8 | 3 | 0 |
| 2-9% PD-L1+ IC | 16 | 11 | 3 | 1 | 0 | 0 |
| 10-49% PD-L1+ IC | 9 | 6 | 3 | 1 | 0 | 0 |
| >50% PD-L1 IC | 1 | 1 | 0 | 0 | 0 | 0 |

| Number at risk |  |  |  |  |  |  |
| --- | --- | --- | --- | --- | --- | --- |
| 0-1% PD-L1+ IC | 42 | 26 | 12 | 2 | 2 | 0 |
| 2-9% PD-L1+ IC | 17 | 10 | 5 | 1 | 0 | 0 |
| 10-49% PD-L1+ IC | 4 | 3 | 0 | 0 | 0 | 0 |
| >50% PD-L1 IC | 1 | 1 | 0 | 0 | 0 | 0 |

**(C) (D)**

**
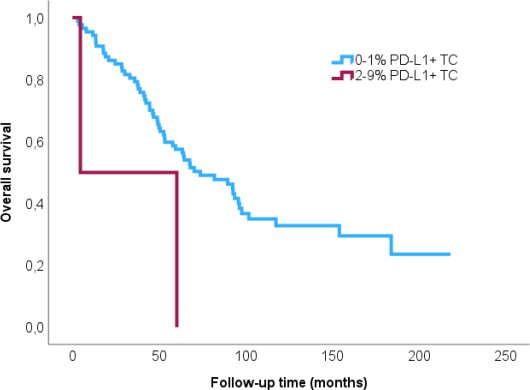

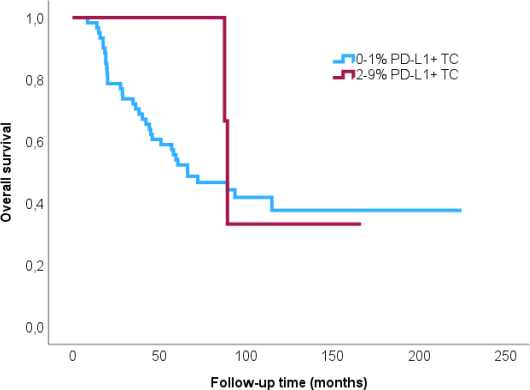
**

| Number at risk |  |  |  |  |  |  |
| --- | --- | --- | --- | --- | --- | --- |
| 0-1% PD-L1+ TC | 63 | 39 | 15 | 8 | 3 | 0 |
| 2-9% PD-L1+ TC | 16 | 11 | 3 | 1 | 0 | 0 |

| Number at risk |  |  |  |  |  |  |
| --- | --- | --- | --- | --- | --- | --- |
| 0-1% PD-L1+ TC | 63 | 39 | 15 | 8 | 3 | 0 |
| 2-9% PD-L1+ TC | 16 | 11 | 3 | 1 | 0 | 0 |

**(E) (F)**

**
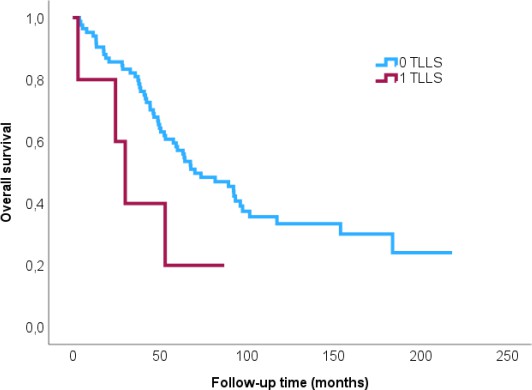

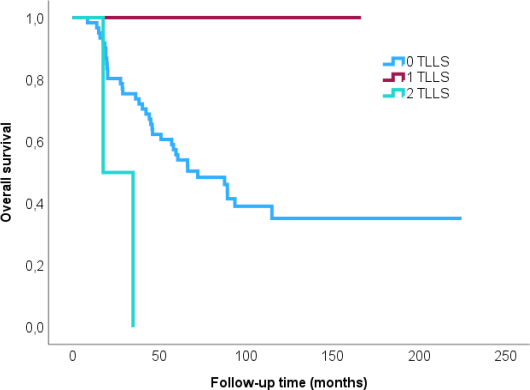
**

| Number at risk |  |  |  |  |  |  |
| --- | --- | --- | --- | --- | --- | --- |
| 0 TLLS | 84 | 54 | 21 | 10 | 3 | 0 |
| 1 TLLS | 5 | 2 | 0 | 0 | 0 | 0 |

| Number at risk |  |  |  |  |  |  |
| --- | --- | --- | --- | --- | --- | --- |
| 0 TLLS | 61 | 38 | 15 | 2 | 2 | 0 |
| 1 TLLS | 2 | 2 | 1 | 0 | 0 | 0 |
| 2 TLLS | 2 | 0 | 0 | 0 | 0 | 0 |

**
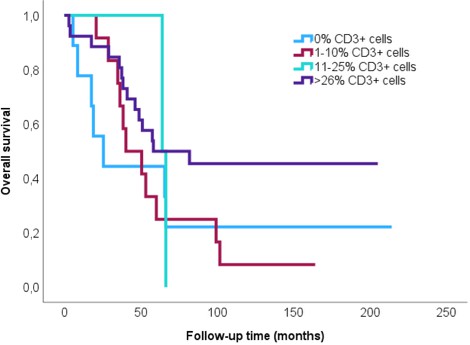

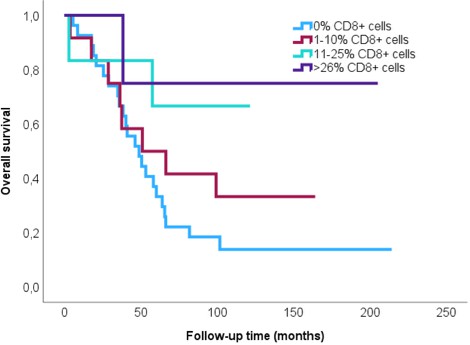
****(A) (B)**

Supplementary Figure 8

| Number at risk |  |  |  |  |  |  |
| --- | --- | --- | --- | --- | --- | --- |
| 0% CD3+ cell s | 9 | 4 | 2 | 0 | 0 | 0 |
| 1-10% CD3+ cell s | 12 | 6 | 2 | 1 | 0 | 0 |
| 11-25% CD3+ cell s | 2 | 2 | 0 | 0 | 0 | 0 |
| >26% CD3+ cel ls | 26 | 16 | 8 | 3 | 1 | 0 |

| Number at risk |  |  |  |  |  |  |
| --- | --- | --- | --- | --- | --- | --- |
| 0% CD8+ cell s | 27 | 13 | 4 | 1 | 1 | 0 |
| 1-10% CD8+ cell s | 12 | 7 | 4 | 2 | 0 | 0 |
| 11-25% CD8+ cell s | 6 | 5 | 1 | 0 | 0 | 0 |
| >26% CD8+ cel ls | 4 | 3 | 3 | 2 | 1 | 0 |

**
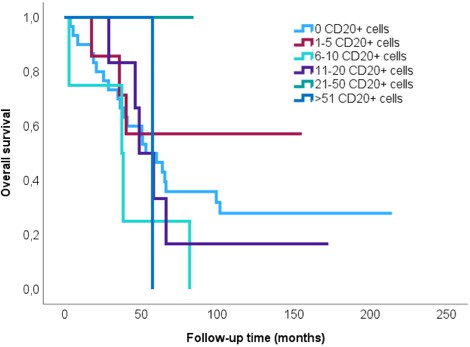

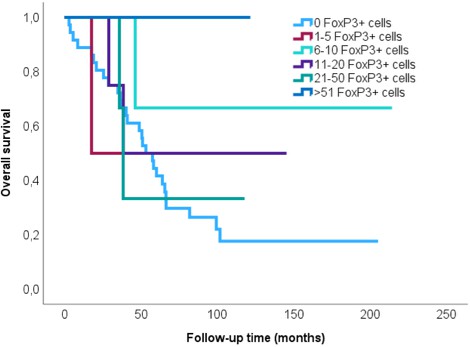
(C) (D)**

| Number at risk |  |  |  |  |  |  |
| --- | --- | --- | --- | --- | --- | --- |
| 0 CD20+ cells | 30 | 18 | 8 | 3 | 2 | 0 |
| 1-5 CD20+ cells | 7 | 4 | 3 | 1 | 0 | 0 |
| 6-10 CD20+ cells | 4 | 1 | 0 | 0 | 0 | 0 |
| 11-20 CD20+ cells | 6 | 3 | 1 | 1 | 0 | 0 |
| 21-50 CD20+ cells | 1 | 1 | 0 | 0 | 0 | 0 |
| >51 CD20+ cells | 1 | 1 | 0 | 0 | 0 | 0 |

| Number at risk |  |  |  |  |  |  |
| --- | --- | --- | --- | --- | --- | --- |
| 0 Fox P3+ cel ls | 36 | 21 | 5 | 3 | 1 | 0 |
| 1-5 Fox P3+ cel ls | 2 | 1 | 1 | 0 | 0 | 0 |
| 6-10 Fox P3+ cel ls | 3 | 2 | 2 | 2 | 1 | 0 |
| 11-20 Fox P3+ cel ls | 4 | 2 | 2 | 0 | 0 | 0 |
| 21-50 Fox P3+ cel ls | 3 | 1 | 1 | 0 | 0 | 0 |
| >51 Fox P3+ cells | 1 | 1 | 1 | 0 | 0 | 0 |

**
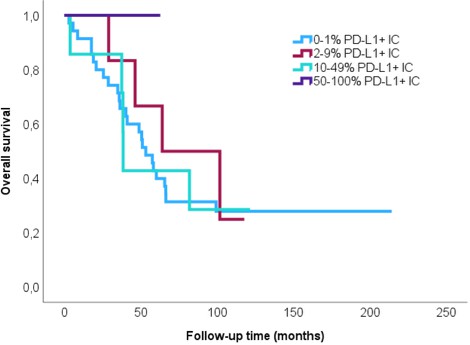

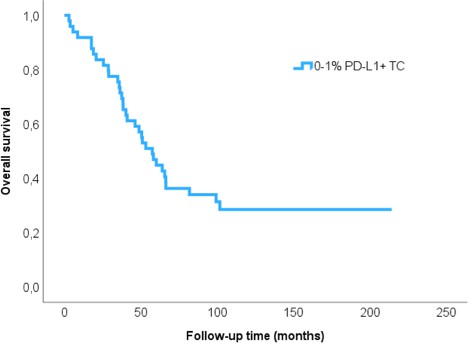
(E) (F)**

| Number at risk |  |  |  |  |  |  |
| --- | --- | --- | --- | --- | --- | --- |
| 0-1% P D-L1+ IC | 35 | 20 | 8 | 5 | 2 | 0 |
| 2-9% P D-L1+ IC | 6 | 4 | 2 | 0 | 0 | 0 |
| 10-49% P D-L1+ IC | 7 | 3 | 2 | 0 | 0 | 0 |
| 50-100% P D-L1+ IC | 1 | 1 | 0 | 0 | 0 | 0 |

| Number at risk |  |  |  |  |  |  |
| --- | --- | --- | --- | --- | --- | --- |
| 0-1% P D-L1+ TC | 49 | 28 | 12 | 5 | 2 | 0 |

**(G)**

**
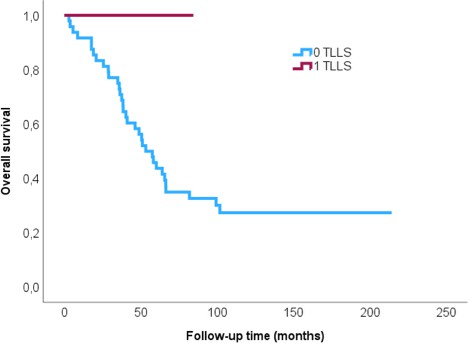
**

| Number at risk |  |  |  |  |  |  |
| --- | --- | --- | --- | --- | --- | --- |
| 0 TLLS | 48 | 27 | 12 | 5 | 2 | 0 |
| 1 TLLS | 1 | 1 | 0 | 0 | 0 | 0 |
